# Integrative in vitro, in vivo, and in silico investigation with mineral profiling of *Combretum indicum*: A multi-target therapeutic candidate exhibiting antioxidant, thrombolytic, anti-inflammatory, anti-arthritic, analgesic, cytotoxic, and anthelmintic activities

**DOI:** 10.1371/journal.pone.0360173

**Published:** 2026-10-26

**Authors:** Zobayed Islam, Hridoy Chowdhury, Sujan Kanti Das, Ahnuf Zaman Tomal, Anik Das, S. M. Naim Uddin

**Affiliations:** 1 Department of Pharmacy, Faculty of Biological Science, University of Chittagong, Chattogram, Bangladesh; 2 Department of Pharmacy, BGC Trust University Bangladesh, Chittagong, Bangladesh; 3 Bangladesh Council of Scientific and Industrial Research, Chattogram, Bangladesh; Guru Nanak College, INDIA

## Abstract

This study aimed to comprehensively evaluate the pharmacological and computational potential of the methanolic extract of *Combretum indicum*. Mineral profiling by ICP–AES confirmed the presence of essential elements, including K (5.92 ppm), Mg (4.94 ppm), Fe (1.92 ppm), and Zn (1.13 ppm), with no detectable toxic heavy metals. The extract exhibited strong antioxidant activity with 95.48% inhibition of H_2_O_2_ and an IC_50_ of 0.82 µg/mL. Significant thrombolytic activity was observed, achieving 82.71% clot lysis, exceeding streptokinase. Anti-inflammatory activity showed 95.27% inhibition in vitro and 65% edema reduction in vivo. Anti-arthritic activity reached 85.6% inhibition in vitro and 87.50% in vivo, surpassing indomethacin. Analgesic activity demonstrated up to 70.15% inhibition of writhing. Cytotoxicity (LC_50_ = 30.19 µg/mL) and strong anthelmintic effects further supported bioactivity. Molecular docking revealed high binding affinities (up to −10.6 kcal/mol), while ADME and drug-likeness analyses indicated favorable pharmacokinetic profiles. DFT analysis confirmed chemical stability and reactivity. Overall, *C. indicum* demonstrated promising multi-target therapeutic potential, warranting further investigation for drug development.

## Introduction

Medicinal plants have long served as a cornerstone of therapeutic development, contributing significantly to both traditional and modern healthcare systems [1]. A substantial proportion of currently approved drugs are either directly derived from plant sources or inspired by phytochemical scaffolds, underscoring the enduring importance of botanical resources in drug discovery [2, 3]. The increasing prevalence of chronic and multifactorial diseases—such as cardiovascular disorders, inflammatory conditions [4], cancer [5], and metabolic syndromes [6]—has intensified the demand for safer, multi-target therapeutic agents. In this context, plant-derived bioactive compounds offer distinct advantages due to their structural diversity, biological compatibility, and ability to modulate multiple biochemical pathways simultaneously [2,7].

Among medicinal plants, *Combretum indicum* (L), belonging to the family Combretaceae occupied a distinct place among medicinal plants, has attracted attention due to its wide distribution and extensive utilization in traditional medicine across tropical and subtropical regions [8]. The plant has been historically utilized for the treatment of parasitic infections, inflammation, gastrointestinal disorders, and microbial diseases. Phytochemical investigations have revealed the presence of diverse secondary metabolites, including flavonoids, tannins, alkaloids, glycosides, and phenolic compounds, which are known to exhibit antioxidant, anti-inflammatory, antimicrobial, and cytotoxic activities. Despite these promising attributes, existing studies on *C. indicum* remain fragmented and largely focused on isolated pharmacological effects [8, 9]. A systematic and integrative evaluation encompassing multiple biological activities along with mechanistic insights is still lacking, highlighting a clear research gap.

One crucial aspect in medicinal plant research, yet often overlooked, is the elemental composition, which can significantly influence both therapeutic efficacy and safety [10]. Essential macro- and micro-elements such as calcium, magnesium, potassium, iron, and zinc play vital roles in physiological processes, including enzyme activation, cellular signaling, and redox balance. Conversely, the presence of toxic heavy metals may pose serious health risks [11, 12]. Therefore, the determination of mineral content using advanced analytical techniques such as Inductively Coupled Plasma–Atomic Emission Spectroscopy (ICP–AES) is essential to validate the nutritional and toxicological profile of plant materials and ensure their safe medicinal application [13, 14].

Oxidative stress is a fundamental pathological mechanism underlying numerous ailments, resulting from an imbalance between reactive oxygen species (ROS) generation and antioxidant defense systems. Hydrogen peroxide (H_2_O_2_), a relatively stable ROS, can penetrate biological membranes and generate highly reactive hydroxyl radicals, leading to cellular damage. Consequently, the evaluation of in vitro antioxidant activity using H_2_O_2_ scavenging assays provides valuable insight into the free radical neutralizing capacity of plant extracts and their potential to mitigate oxidative stress–related disorders [15, 16].

Thrombotic disorders represent another major global health concern, contributing significantly to cardiovascular morbidity and mortality. The formation of abnormal blood clots can obstruct blood vessels and lead to life-threatening conditions such as myocardial infarction and stroke. Although synthetic thrombolytic agents such as streptokinase are widely used, they are often associated with adverse effects and high cost. Therefore, the exploration of plant-based thrombolytic agents through in vitro clot lysis assays offers a promising alternative approach for identifying safer and more accessible therapeutic options [17–19].

Inflammation is a complex biological response that plays a central role in the pathogenesis of various chronic diseases. While acute inflammation is protective, its chronic persistence can lead to tissue damage and disease progression. The evaluation of anti-inflammatory activity through both in vitro protein denaturation assays and in vivo carrageenan-induced paw edema models provides comprehensive insights into the ability of bioactive compounds to stabilize proteins, inhibit inflammatory mediators, and suppress edema formation [20–22].

Arthritis, which is closely related to inflammation, is a debilitating condition characterized by joint inflammation, pain, and structural damage [23]. The development of effective anti-arthritic agents requires both in vitro and in vivo validation to assess their capacity to prevent protein denaturation and reduce chronic inflammatory responses [24]. Experimental models such as bovine serum albumin denaturation assays and formaldehyde-induced arthritis models are widely employed to evaluate the therapeutic potential of plant extracts in managing arthritic conditions [25, 26].

Pain management remains a critical component of clinical treatment, particularly in inflammatory and degenerative diseases. The assessment of analgesic activity using in vivo models such as the acetic acid–induced writhing test allows for the evaluation of peripheral antinociceptive effects mediated through the inhibition of inflammatory mediators such as prostaglandins [20, 21].

In addition to therapeutic efficacy, the cytotoxic potential of plant extracts is an important parameter for preliminary screening of bioactive compounds with possible anticancer properties. The brine shrimp lethality assay, employing *Artemia salina*, is a rapid and cost-effective method widely used to evaluate general cytotoxicity and identify compounds with potential pharmacological relevance [5,27].

Recent advances in computational biology have revolutionized the drug discovery process by enabling the prediction of molecular interactions and pharmacokinetic properties prior to experimental validation [28]. In silico approaches, including ligand selection, target identification, molecular docking, and visualization, provide detailed insights into the binding affinity and interaction patterns of bioactive compounds with specific protein targets. These methods facilitate the identification of promising lead compounds and help elucidate their mechanisms of action at the molecular level [29, 30].

Furthermore, the evaluation of pharmacokinetic parameters through ADME (Absorption, Distribution, Metabolism, and Excretion) analysis is essential for determining the drug-likeness and bioavailability of candidate molecules [31]. Complementary assessments of drug-likeness properties, based on established criteria such as Lipinski’s rule of five, along with toxicity prediction, are critical for identifying compounds with favorable safety and efficacy profiles [32–34].

Density Functional Theory (DFT) calculations provide a deep understanding of electronic properties, stability, and reactivity of molecules by analyzing parameters such as frontier molecular orbitals (HOMO–LUMO), energy gap, and dipole moment. These quantum chemical descriptors play a crucial role in understanding the structure–activity relationships and guiding the rational design of novel therapeutic agents [35–37].

In light of these considerations, the present study aims to address the existing research gap by conducting a comprehensive pharmacological and computational investigation of *Combretum indicum*. The study integrates mineral content analysis, in vitro antioxidant and thrombolytic assays, in vitro and in vivo anti-inflammatory and anti-arthritic evaluations, analgesic and cytotoxic assessments, along with advanced in silico modeling, ADME profiling, drug-likeness prediction, toxicity analysis, and DFT calculations. This multi-level approach is expected to provide a holistic understanding of the therapeutic potential of *C. indicum* and support its development as a promising source of novel, multi-target pharmacological agents suitable for future drug discovery initiatives.

## Materials and methods: Plant collection, identification, and crude extract preparation

The whole plant of *Combretum indicum* was collected from the Botanical Garden of the University of Chittagong during its active growth phase. Taxonomic identification was performed and authenticated by Mr. Faruq Ahmed, a qualified taxonomist from the Department of Botany, and a voucher specimen was preserved for future reference. The plant material was washed, shade-dried, and finely powdered. The powdered sample was extracted using methanol through cold maceration, followed by filtration and concentration under reduced pressure using a rotary evaporator. The resulting methanolic crude extract was collected for subsequent pharmacological investigations.

### Determination of mineral content byCP–AESI

The mineral composition of *Combretum indicum* was analyzed using Inductively Coupled Plasma–Atomic Emission Spectroscopy (ICP–AES). About 0.5 g of the dried and pulverized plant material was subjected to dry digestion with concentrated nitric (HNO_3_) and perchloric (HClO_4_) acids by the controlled heating until a clear solution appears. The digest was allowed to cool, filtered, and diluted to volume with deionized water. The resulting solution was analyzed by ICP–AES to quantify the elemental concentrations of Ca, Mg, Na, K, Cu, Fe, Zn, Pd, Cd, Cr, and Ni based on their characteristic emission wavelengths in the plasma.Analyses were performed in triplicates and calibration was carried out by using certified multi-element standard solutions. Concentrations of the elements were expressed in ppm (parts per million) [10,12,14].

### In vitro antioxidant activity via (H_2_O_2_) scavenging assay hydrogen peroxide (H₂O₂) Scavenging Assay

The hydrogen peroxide (H_2_O_2_) scavenging activity of *Combretum indicum* extracts was evaluated to determine their antioxidant potential using a modified spectrophotometric method. Briefly, 0.1 mL of each plant extract was mixed with 3.4 mL of 0.1 M phosphate buffer (pH 7.4) followed by the addition of 0.6 mL of 40 mM H_2_O_2_ solution. The reaction mixture was gently vortexed and incubated for 10 minutes at ambient laboratory temperature to facilitate interaction between reactive oxygen species and the phytochemical constituents present in the extract. After incubation, the absorbance of the mixture was recorded at 230 nm using a UV–visible spectrophotometer against a reagent blank containing phosphate buffer without the test sample. Ascorbic acid was employed as the reference antioxidant standard to allow comparative assessment of radical scavenging efficiency. The capacity of the extracts to neutralize hydrogen peroxide was quantified as percentage inhibition relative to the control according to the following equation:


$$\begin{array}{c} {𝐇}_{2}{𝐎}_{2}\;𝐬𝐜𝐚𝐯𝐞𝐧𝐠𝐢𝐧𝐠\;(\%)=\frac{A_{0}-A_{1}}{A_{0}}\times 100 \end{array}$$

where (A_0_) represents the absorbance of the control reaction (containing all reagents except the extract), and (A_1_) denotes the absorbance measured in the presence of the plant extract. This assay reflects the ability of bioactive phytoconstituents in *Combretum indicum* to detoxify hydrogen peroxide, thereby preventing the generation of highly reactive hydroxyl radicals and mitigating oxidative stress [16].

### In vitro thrombolytic (clot lysis) assay

The thrombolytic potential of the methanolic extract of *Combretum indicum* was evaluated using an in vitro clot lysis model following the method originally described by R. Prasad and colleagues with slight modifications. Fresh venous blood samples were collected from ten healthy adult volunteers (n = 10) who had no prior history of anticoagulant therapy or oral contraceptive use. Approximately 5 mL of blood was aseptically drawn from each volunteer and immediately distributed into ten previously weighed sterile microcentrifuge tubes (0.5 mL per tube). The tubes were then incubated at 37 °C for 45 minutes to facilitate complete clot formation.Following clot formation, the serum was carefully aspirated without disturbing the underlying clot, and each tube was reweighed to determine the net clot weight. Subsequently, 100 μL of the methanolic extract of *Combretum indicum* (10 mg/mL) was added to each microcentrifuge tube containing the pre-weighed clot. For comparative evaluation, 100 μL of Streptokinase was used as the positive control owing to its well-established fibrinolytic activity, whereas 100 μL of sterile distilled water served as the negative control. All tubes were incubated again at 37 °C for 90 minutes to allow clot dissolution. After incubation, the released fluid was carefully removed, and the tubes were reweighed to determine the extent of clot degradation. The difference in weight before and after treatment corresponded to the amount of clot lysed. The thrombolytic activity was expressed as the percentage of clot lysis, calculated based on the reduction in clot weight relative to the initial clot mass. This experimental approach provides a quantitative assessment of the fibrinolytic potential of phytoconstituents present in *Combretum indicum*, which may contribute to its prospective therapeutic value in the management of thrombotic disorders [18, 19].

### Evaluation of anti-inflammatory activity: Protein denaturation assay

The anti-inflammatory capability of *Combretum indicum* extract was evaluated using the in vitro protein denaturation inhibition test, a method widely employed for screening anti-inflammatory agents. This test depends on the fact that protein denaturation, whether from heating or chemical agents, is closely related to inflammation pathophysiology.Compounds capable of halting or reducing protein denaturation are thus thought to be potentially anti-inflammatory by stabilizing protein structure itself. In the present work, the reaction mixtures were obtained by treating 0.45 mL of 5% (w/v) bovine serum albumin (BSA) plus 0.05 mL of various concentrations the plant extract (100–500 µg/mL). To ensure optimal conditions for the assay, the pH of the reaction system was carefully adjusted to 6.3 using 1 N hydrochloric acid (HCl). All the reaction mixtures were then incubated first at 37 °C for 20 minutes in order to allow the protein substrate to adequately interact with bioactive constitutents present in the extract. Subsequently, the samples of mixture were put under thermal induction at 57 °C for 3 minutes in contrast to the gradual rise in temperature during controlled protein denaturation.After thermal treatment, the reaction mixtures were allowed to cool down slowly to ambient temperature and the absorbance was measured at 660 nm by means of a UV-visible spectrophotometer. Diclofenac sodium served as the reference standard anti-inflammatory drug, while the control was a reaction mixture containing distilled water in place of plant extract [24, 38].

The percentage inhibition of protein denaturation was determined according to the following expression:


$$\begin{array}{c} 𝐏𝐞𝐫𝐜𝐞𝐧𝐭𝐚𝐠𝐞\;𝐈𝐧𝐡𝐢𝐛𝐢𝐭𝐢𝐨𝐧\;(\%)=\frac{A_{c}-A_{s}}{A_{c}}\times 100\; \end{array}$$

where *Ac* represents the absorbance of the control reaction and *As* denotes the absorbance of the test sample containing the plant extract.

### In vivo anti-inflammatory activity

The anti-inflammatory activity of Combretum indicum extracts was researched in rats through an inflammation model: carrageenan-induced paw edema. This method works by inducing the release of inflammatory mediators such as histamine, serotonin, bradykinin and prostaglandins. The rats were divided into eight groups (n = 4). One group served as the negative control and received 1% PGA, while another group received 10 mg/kg diclofenac sodium as the standard drug. The remaining groups were treated with different fractions of the plant extract (water, ethyl acetate, and n-hexane) at doses of 50 and 100 mg/kg.30 minutes later, 0.1 ml of carrageenan is injected into the hind paw. Paw swelling was measured at 1, 3 and 6 h. If there was a reduction in paw edema compared to the control group, this indicated that the extracts have notable anti-inflammatory activity [24,39].

### In vitro anti-arthritic activity: Inhibition of protein denaturation using bovine serum albumin

The anti-arthritic activity of the methanolic extract of *Combretum indicum* was evaluated using the bovine serum albumin (BSA) denaturation assay [40], a common in vitro method for assessing anti-inflammatory potential. Protein denaturation plays a key role in arthritis, and compounds that prevent this process may help manage the disease. The reaction mixture contained BSA and different concentrations of the plant extract (12.5–800 µg/mL). The pH was adjusted to 6.3, followed by incubation at 37 °C for 20 minutes and heating at 57 °C for 30 minutes to induce protein denaturation. After cooling, phosphate-buffered saline was added, and turbidity was measured at 660 nm using a spectrophotometer. Aspirin was used as a standard drug for comparison. Appropriate control samples were also prepared. A decrease in turbidity compared to control indicated that the extract has significant protein-stabilizing and anti-arthritic activity [41]. The percentage inhibition of protein denaturation was calculated according to the following equation:


$$\begin{array}{c} 𝐈𝐧𝐡𝐢𝐛𝐢𝐭𝐢𝐨𝐧\;(\%)=\frac{100-\left(A-B\right)}{C}\times 100 \end{array}$$

where *A* represents the absorbance of the test sample containing the plant extract, *B* corresponds to the absorbance of the product control, and *C* denotes the absorbance of the test control. The magnitude of inhibition reflects the ability of phytoconstituents present in *Combretum indicum* to stabilize protein structures against denaturation, thereby indicating its potential anti-arthritic activity [42, 43].

### In vivo anti-arthritic activity: Formaldehyde-induced arthritis model

The anti-arthritic activity of the methanolic extract of *Combretum indicum* was evaluated using the formaldehyde-induced arthritis model in rats, which closely mimics chronic joint inflammation.

Rats were divided into groups (n = 5). The normal control received distilled water, while the standard group was treated with aspirin (100 mg/kg). The test groups received the extract at doses of 200 and 400 mg/kg orally for 10 days. Arthritis was induced by injecting 0.1 mL of 2% formaldehyde into the hind paw on day 1, followed by a second injection on day 3.

Paw swelling was measured using a digital caliper over the 10-day period. The increase in paw size indicated inflammation, and reduction in swelling in treated groups was compared to the arthritic control. A significant decrease in paw edema suggested that the extract possesses notable anti-arthritic activity and may help reduce chronic inflammation [44, 45].

### Evaluation of analgesic activity using the acetic acid–induced writhing test

The antinociceptive activity of *Combretum indicum* extract was evaluated using the acetic acid–induced writhing test in mice, a standard method for assessing peripheral analgesic effects. This model induces pain by releasing inflammatory mediators such as prostaglandins, leading to a characteristic writhing response [46].Swiss albino mice (20–22 g) were divided into groups. The control group received normal saline, while the standard group was treated with diclofenac sodium(10 mg/kg). The test groups were administered the plant extract orally at doses of 200 and 400 mg/kg. After 30 minutes, pain was induced by intraperitoneal injection of 1% acetic acid. Following a 5-minute delay, the number of writhing responses—marked by abdominal contractions and hind limb stretching—was counted for 10 minutes. A reduction in the number of writhes compared to the control group indicated significant analgesic activity of the extract [47]. The analgesic efficacy of the plant extract was determined by calculating the percentage inhibition of writhing relative to the control group using the following equation:


$$\begin{array}{c} 𝐏𝐞𝐫𝐜𝐞𝐧𝐭𝐚𝐠𝐞\;𝐢𝐧𝐡𝐢𝐛𝐢𝐭𝐢𝐨𝐧\;(\%)=\;\left(1-\frac{w_{t}}{w_{c}}\right)\times 100 \end{array}$$

where *W_t_* represents the mean number of writhes observed in the treated group, and *W_C_* denotes the mean number of writhes recorded in the control group [48].

### Evaluation of cytotoxic activity using brine shrimp (*Artemia salina*) lethality bioassay

In experiment, they employ the brine shrimp lethality assay -- a relatively simple and cost-effective means to test for active compounds of drugs. Ready unfertilized eggs of *Artemia salina* are placed into artificial seawater (3.8% NaCl) to mature for 48 hours and to hatch into nauplii. Serial dilutions of the extract (6.25–400 μg/mL) were prepared in DMSO. DMSO in artificial seawater served as the negative control, whereas vincristine sulfate was used as the positive control. Some 20 nauplii were added to each test tube with these substances. After 24 hours of incubation, the number of surviving nauplii was counted and their mortality rate determined. An increased mortality compared with the control group demonstrates that the extract has a considerable degree of cytotoxicity [47].

The percentage of lethality was calculated relative to the total number of nauplii introduced into each vial using the following equation:


$$\begin{array}{c} 𝐌𝐨𝐫𝐭𝐚𝐥𝐢𝐭𝐲\;(\%)=\frac{N_{t}}{N_{0}}\times 100 \end{array}$$

where (*N*_*t*_) represents the number of dead nauplii after 24 hours of exposure and (*N*_*0*_) denotes the initial number of nauplii transferred (20 per vial). The median lethal concentration (LC₅₀), representing the concentration required to cause 50% mortality of the brine shrimp population, was subsequently estimated through regression analysis. This bioassay provides a valuable preliminary indicator of the cytotoxic and potential antitumor properties of phytochemicals present in *Combretum indicum* [48,49].

### In vitro anthelmintic activity assay

The anthelmintic activity of the methanolic extract of *Combretum indicum* was evaluated using an in vitro parasite inhibition assay. Different concentrations of the extract (50–200 mg/mL) were prepared in phosphate-buffered saline (PBS) to observe dose-dependent effects. Albendazole (5 and 10 mg/mL) was used as the standard drug, while parasites in PBS without treatment served as the control. Equal numbers of live parasites were placed in each solution and observed under controlled conditions. The time required for paralysis and death of the parasites was recorded. A decrease in motility and survival time compared to the control group indicated significant anthelmintic activity. These findings suggest that the extract may contain bioactive compounds capable of disrupting the normal function of parasitic worms [50, 51].

### Animal euthanasia

Finally, at the conclusion of all experimental protocols, the animals were subjected to humane euthanasia in accordance with internationally agreed-upon ethical standards designed to cause minimal stress and pain. Euthanasia is achieved by injection of a lethal dose of a suitable anesthetic agent (e.g., a ketamine/xylazine mixture) into the abdominal cavity of the animal. Death is determined by cessation of heart beat and respiratory movement before disposal of the body. All animal handling and experimental protocols were conducted in accordance with standard laboratory animal care and use standards designed to cause minimal stress and pain.

### Ethics statement

All experimental procedures in animal studies were conducted in strict conformity with ethical 333 guidelines and animal care and use guidelines. This research was prospectively approved by the Animal Research Ethics Committee of the Faculty of Biological Sciences, University of Chittagong, Bangladesh, on February 20, 2025. The reference number is AERB-FBSCU-20260220-(2). There was no waiver.The animal suffering was minimized using appropriate methods, such as anesthesia during experimental procedures, and euthanasia was performed using an overdose of anesthetic agents at the termination of the experiment. For the in **vitro thrombolytic assay**, human blood samples were collected from healthy adult volunteers after obtaining written informed consent. This procedure was approved by the same ethics committee (Approval No. **AERB-FBSCU-20260628-01**) and was conducted in accordance with the relevant ethical guidelines for research involving human participants.

### Acute oral toxicity study

Acute oral toxicity tests were carried out on the whole plant extract of *Combretum indicum* using healthy adult Swiss albino mice of either sex with body weights ranging from 20 to 25 g. The tests were carried out following the guidelines set by the Organisation for Economic Co-operation and Development (OECD guideline 423: Acute Oral Toxicity – Acute Toxic Class Method). In this study, the extract was administered orally to the mice at a starting dose level of 2000 mg/kg body weight. The animals were individually observed during the first 30 minutes, periodically during the first 24 hours with special attention during the first 4 hours, and daily for the next 14 days. Parameters such as behavior, physical appearance, food and water consumption, and death rates were monitored. Body weights were also recorded during the course of the experiment.Since no death rates were recorded at the tested dose level, it is evident that the LD50 value of the extract is greater than 2000 mg/kg. Based on this, the extract of *Combretum indicum* can be said to be relatively safe for further pharmacological studies.

### In silico activity ligand selection, target identification, molecular docking, and visualization

The molecular docking study was performed using phytochemicals previously identified by GC–MS analysis of the ethanol extract of *Combretum indicum*, as reported by our research group. Compounds with documented biological relevance and suitable structural information were selected for docking. Their three-dimensional structures were retrieved from the PubChem database in SDF format, converted to PDB format using Open Babel, and energy-minimized prior to docking. The complete GC–MS chromatogram and compound identification data are available in our previous publication [52]. For target identification, the crystal structure of the relevant proteins was obtained from the Protein Data Bank and can be further subdivided by specific PDB IDs: 1DNU (antioxidant) [53], 2HLO (thrombolytic [54]), 1CX2 (anti-inflammatory [55]), 6RMJ (anti-arthritic [56]), 5IKR (analgesic) [57], 1NME (cytotoxic [58]), 1SA0 (anthelmintic [59]). The protein structures were prepared by removing water molecules, co-crystallized ligands and heteroatoms; addition of polar hydrogens as well as Kollman charges with AutoDock Tools. The molecular docking simulations were carried out by AutoDock Vina in order to predict the binding energies between ligands and receptor proteins. The docking protocol was validated by re-docking the co-crystallized ligand into the active site of each receptor. The obtained root mean square deviation (RMSD) values were ≤2.0 Å, confirming the reliability of the docking procedure. Molecular docking simulations were then carried out using AutoDock Vina. Grid boxes were centered on the active sites defined by the co-crystallized ligands, and the corresponding grid center coordinates, dimensions, and docking parameters are summarized in S1 Table. Unless otherwise specified, the exhaustiveness parameter was set to 8. For each ligand, nine binding poses were generated, and the conformation with the lowest binding affinity (kcal/mol) was selected for further analysis.The resulting complexes were visualized, and then analyzed, using Discovery Studio Visualizer as well as PyMOL to identify important interactions such as hydrogen bond formations, Van der Waals forces or π–π stacking (stackings of electron clouds upon one another via Pi-orbital overlap). These findings were then further interpreted in order to understand how this might lead into potential drug discovery strategies appropriate especially under local environments.

#### ADMET and drug-likeness properties analysis.

SwissADME and pkCSM were used to evaluate how well the selected compounds met criteria for drug likeness and the drug’s movements. The following parameters were investigated- Gastrointestinal absorption, blood–brain barrier penetration, cytochrome P450 interactions and potential toxic effects. With Lipinski’s rule of five, as well as other standard criteria to estimate oral bioavailability and so forth, we were able to gauge their success in light of several indices. Through such analyses, it is possible to see whether these compounds possess the appropriate characteristics needed to serve as potential drug candidates, which will help pave their way into further development for use in pharmacology research [29–32,60].

#### Density functional theory.

The electronic structure and regioselectivity of the donor–acceptor (D–A) systems were analyzed using density functional theory (DFT). In addition to frontier molecular orbital analysis, global DFT reactivity descriptors were incorporated to compensate for the limitations of FMO theory in regioselectivity prediction [61]. All calculations were carried out using the hybrid B3LYP density functional method, which provides reliable molecular geometries and electronic energy parameters with good computational efficiency. Full geometry optimizations were performed without imposing any symmetry constraints using the 6-31G(d,p) basis set, as implemented in the Gaussian 09 program. The optimized structures were confirmed to correspond to true energy minima by the absence of imaginary frequencies in the vibrational analysis. Global reactivity descriptors were derived from the frontier molecular orbital energies. The chemical hardness (η), which represents resistance to electron transfer, and the electronic chemical potential (μ), associated with charge-transfer capability, were calculated using the following expressions:


$$\begin{array}{c} \eta =\epsilon \text{LUMO}-\epsilon \text{HOMO} \end{array}$$

$$\begin{array}{c} \mu =\;\frac{\epsilon \text{HOMO}+\epsilon \text{LUMO}}{\text{2}} \end{array}$$

In addition, chemical softness (S), indicating molecular polarizability, and electronegativity (χ), reflecting the tendency to attract electrons, were calculated as:


$$\begin{array}{c} S=\;\frac{\text{1}}{\eta } \end{array}$$

$$\begin{array}{c} \chi =-\mu \end{array}$$

These global reactivity descriptors were employed to evaluate the electronic stability, reactivity, and charge-transfer behavior of the studied molecules and to support the interpretation of their biological interaction potential. These descriptors were used to rationalize the chemical reactivity and regioselectivity of the systems under study. Molecular geometries and orbitals were visualized using GaussView 5.0.9. [62].

### Statistical analysis

All experimental data were expressed as mean ± standard deviation (SD) and analyzed using GraphPad Prism. Statistical significance was determined by one-way analysis of variance (ANOVA) followed by appropriate post hoc tests. A p-value < 0.05 was considered statistically significant, ensuring the reliability and reproducibility of the experimental findings.

## Results and discussion

### Mineral composition of *Combretum indicum* determined by ICP–AES

The elemental composition of Combretum indicum was determined by Inductively Coupled Plasma–Atomic Emission Spectroscopy (ICP–AES), the detected elemental concentrations are listed in Table 1 and shown in Fig 1. The analysis unveiled the existence of several essential macro-and microminerals, but could not detect any heavy metals with potentially toxic characteristics among all substances measured here.

**Table 1:** Elemental composition of *Combretum indicum* determined by Inductively Coupled Plasma–Atomic Emission Spectroscopy (ICP–AES), showing the concentrations of macro-elements, micro-elements, and heavy metals expressed in parts per million (ppm).

Micro-Elements		Macro-Elements		Heavy Metal	
Elements	Amounts (PPM)	Elements	Amounts (PPM)	Elements	Amounts (PPM)
Ca	0.45	Cu	0.0815	Pd	0
Mg	4.94	Cr	0	Cd	0
Na	2.02	Fe	1.92	Cr	0
K	5.92	Zn	1.13	Ni	0

**Fig 1:**
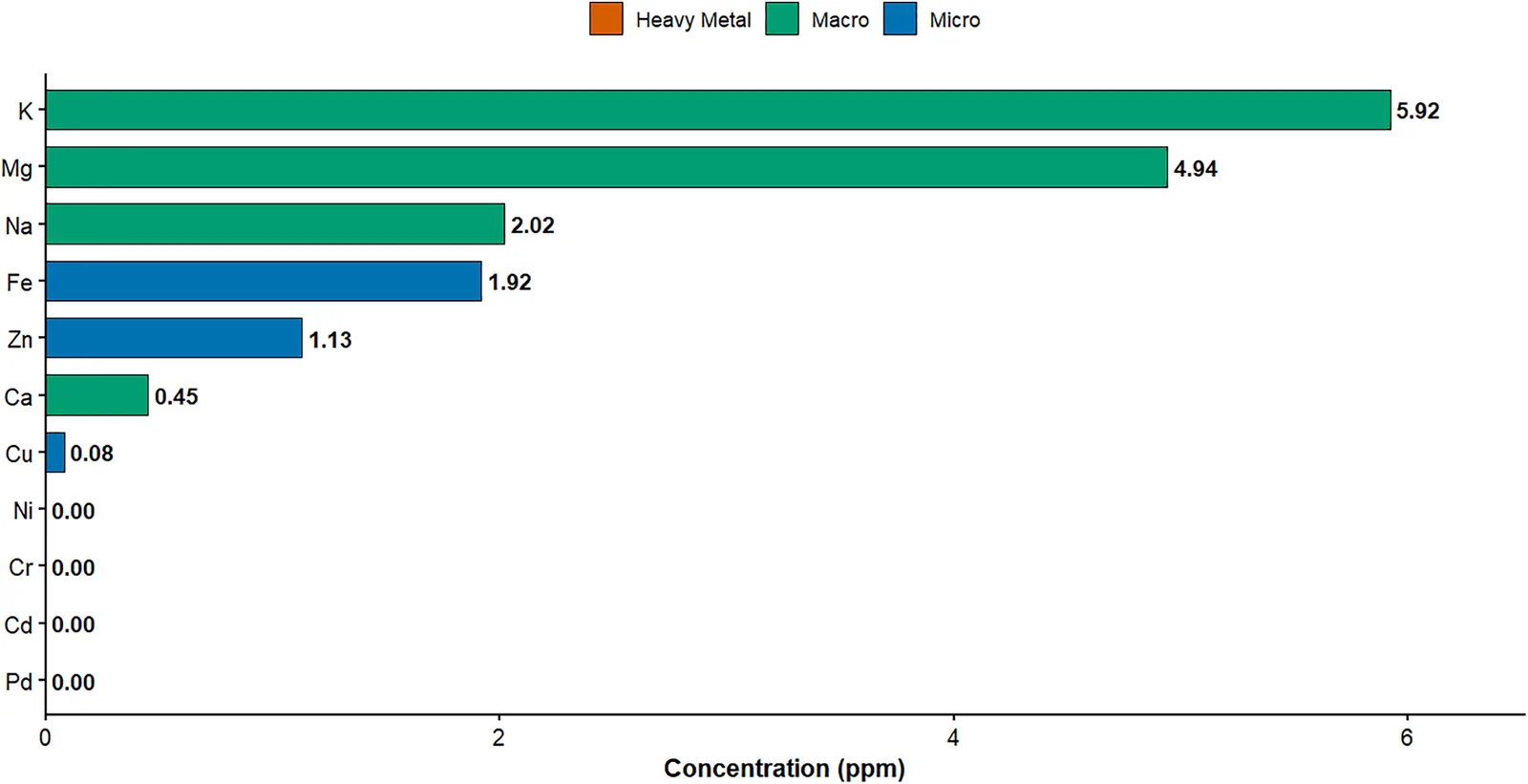
Graphical representation of the mineral profile of *Combretum indicum* obtained from ICP–AES analysis, illustrating the relative abundance of macro-elements, micro-elements, and the absence of detectable heavy metals.

Among the macrominerals potassium (K) was most abundant with a concentration of 5.92 ppm. Magnesium (Mg) scored 4.94 ppm, closely followed by sodium (Na) at 2.02 ppm, then calcium Ca at 0.45 ppm. These macrominerals are well known for their basic physiological roles as components in plant metabolism and human nutrition including regulation of electrolyte balance, activation of enzymes, signal transductions between cells... The relatively higher levels of potassium and magnesium suggest that *C. indicum* is able to provide some of the nutritional and pharmacological benefits credited to this medicinal species.

Of the microminerals the highest level measured was for iron (Fe) at 1.92 ppm. This was followed by zinc (Zn) of no less than 1.13 ppm and then copper (Cu) at 0.0815 ppm. These trace elements play an essential role in the mechanisms of many redox enzymes and metabolic pathways. Their overall presence undoubtedly helps account for *C. indicum’s* therapeutic potential, specifically its ability to provide resistance against oxidative attack as well as preserve the activities of enzymes.

In contrast, heavy metals such as palladium (Pd), cadmium (Cd), chromium (Cr) and nickel (Ni) were all absent (0 ppm) from that analyzed sample. This assures us that the plant material used here is environmentally safe and not polluted with any toxic elements.

The result of ICP–AES profile shows that *Combretum indicum* provides a broad spectrum of mineral elements and elements relevant for nutrition or pharmacology but that it remains free from detectable heavy metals Fig 1 gives a graphic representation of the relative abundance of these elements received in sample while Table 1 displays their distribution in detail.

### Evaluation of antioxidant activity

The antioxidant potential of the *Combretum indicum* extract was evaluated using the DPPH free radical scavenging assay. The extract demonstrated a concentration-dependent increase in radical scavenging activity across the tested concentration range (31.25–1000 μg/mL). As presented in Table 2, the extract exhibited 32.93% inhibition at 31.25 μg/mL, which progressively increased to 42.99%, 60.11%, 78.11%, 80.11%, and 94.73% at concentrations of 62.5, 125, 250, 500, and 1000 μg/mL, respectively. The highest scavenging activity (94.73%) was observed at 1000 μg/mL, indicating a strong free radical neutralizing capacity at higher concentrations.

**Table 2:** Antioxidant activity of *Combretum indicum* extract at different concentrations expressed as percentage inhibition of free radicals.

Serial no.	Absorbance of blank	Conc.(μg/ml)	Absorbance of extract	Inhibition (%)	IC_50_( μg/mL)
1.	0.602	31.25	0.421	32.929	59.36
2.		62.5	0.241	42.99287	
3.		125	0.157	60.10638	
4.		250	0.151	78.10651	
5.		500	0.136	80.11254	
6.		1000	0.124	94.73203	

The calculated IC₅₀ value of the *C. indicum* extract was 59.36 μg/mL, whereas the standard antioxidant exhibited a substantially lower IC₅₀ value of 2.04 μg/mL, confirming its greater antioxidant potency. Nevertheless, the extract displayed considerable DPPH radical scavenging activity, suggesting the presence of bioactive phytochemicals capable of donating hydrogen atoms or electrons to neutralize free radicals. The concentration-dependent inhibition and IC₅₀ values are summarized in Table 2, while the dose-response relationship is illustrated in Fig 2.

**Fig 2:**
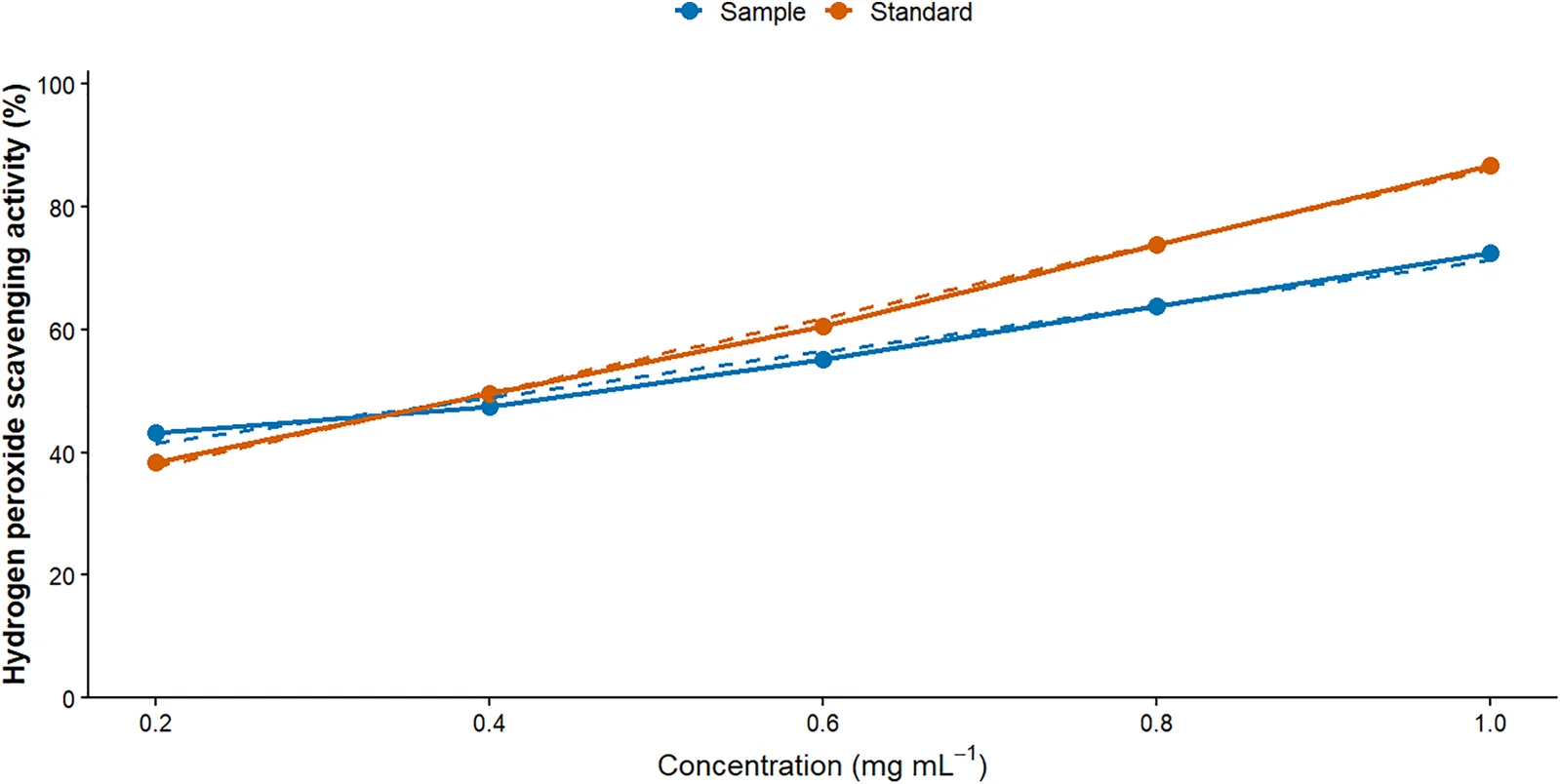
Dose–response curve showing the concentration-dependent antioxidant activity of *Combretum indicum* extract.

### Evaluation of thrombolytic activity

The in vitro clot lysis assay using a methanolic extract of *Combretum indicum*, designated as MECI, presented the outcomes in Table 3, which is reproduced from among them in Fig 3. The assay proved that the plant extract contained a powerful thrombolytic substance and so could possibly have implications for preventing blood clots and thrombotic diseases. The negative control group proved with minimal clot lysis activity a mean value of 17.77 ± 13.70, which suggests that untreated conditions do not engender significant thrombolytic action. In contrast, the standard thrombolytic agent, Streptokinase, caused a much greater clot lysis percentage at 88.98 ± 6.22; this shows that our model was sensitive and reliable experimentally.The methanolic extract of *Combretum indicum (MECI)* produced a significant thrombolytic effect, which was higher than that of the control group (P < 0.01). The highest clot lysis activity was observed at the MECI – 20 mg/mL level, with a mean of 82.71 ± 6.25, well above the standard drug at the same concentration (P < 0.01). Also, MECI – 10 mg/mL had good thrombolytic effects, with an average of 75.23 ± 10.71; even MECI – 05 mg/mL or MECI – 2.5 mg/mL showed significant clot-lysis capabilities which gave respective averages (all unchanged) 70.36 ± 8.24 and 68.08 ± 9.12. The results show an increasingly striking clot semifluidization with growing quantity of extract, indicating that the effect is potent and Thus, we venture to suggest that bioactive components of *Combretum indicum* may help blood clots dissolve or help accelerate the self-destruction of fibrin networks within those clots. The results summarized in Table 3 reveal MECI is much more active in terms of thrombolysis than the control group, while Fig 3 provides clear proof that MECI acts dose-dependently to dislodge blood clots. These results strongly suggest that *Combretum indicum* contains phytochemicals with potential fibrinolytic or anticoagulant properties, which could make it an effective adjunctive therapy in the prevention or management of thrombotic cardiovascular diseases. To sum up, the data described above show that *Combretum indicum* has powerful thrombolytic properties and thus calls for further research into natural thrombolytic agents among its bioactive contents.

**Table 3:** *In vitro* thrombolytic activity of the methanolic extract of *Combretum indicum* (MECI) at different concentrations. The data are expressed as mean ±  SEM representing the percentage of clot lysis compared with the control group. The standard thrombolytic agent Streptokinase was used as the positive control. Statistical significance was determined relative to the control group (p < 0. 01).

Concentration(μg/mL)	MEAN±SEM
Control	17.772 ± 13.70
Standard	88.98 ± 6.22
MECI-20	82.71 ± 6.25**
MECI-10	75.23 ± 10.71**
MECI-05	70.36 ± 8.24**
MECI-2.5	68.08 ± 9.12**

**Fig 3:**
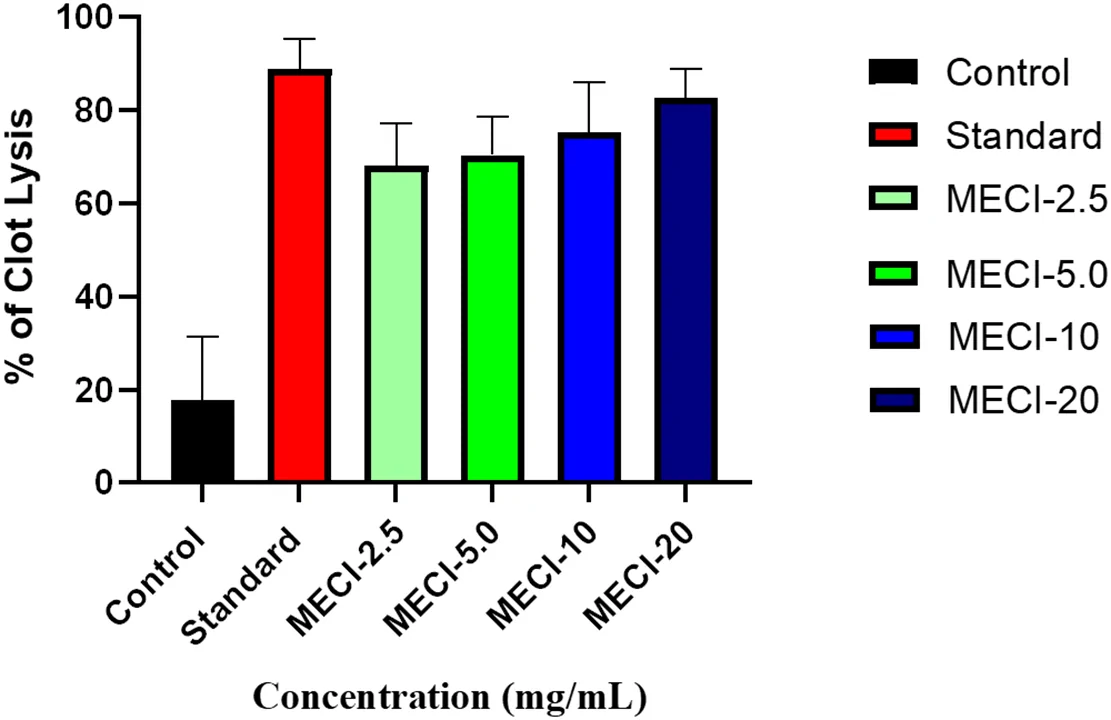
Graphical representation of the concentration-dependent thrombolytic activity of the methanolic extract of *Combretum indicum* (MECI) compared with the control and the standard drug Streptokinase. The extract exhibited significant clot lysis activity, with the highest thrombolytic effect observed at 20 mg/mL, indicating the potential presence of bioactive phytoconstituents capable of promoting fibrinolytic activity.

### Evaluation of in vitro and in vivo anti-inflammatory activity

#### In vitro protein denaturation assay.

The methanolic extract of Combretum indicum (MECI) was tested for its anti-inflammatory activity using the protein denaturation inhibition assay, a well-known in vitro model that is used to look for compounds capable of keeping inflammation’s destabilization of protective proteins around. The results were presented in Table 4, showing that at each tested concentration MECI exhibited a dose-dependent inhibitory action on protein denaturation and thus accumulation within the scope examined here (31.25–1000 μg/mL range). In column four of Table 4, MECI was shown to be more and more effective at inhibiting protein denaturation as its concentration climbed. The highest inhibition (95.27%) of protein denaturation rose up at 1000 μg/mL, while the lowest concentration tests wholesaled processed (20.83% for 31.25 µg/mL). At trail concentrations of 62.5, 125, 250 and 500 µg/mL, this trend continued: 37.50%, 63.19%, 74.30% and 83.33% respectively. The IC_50_ was calculated as 132.49 μg/mL, which is quite high and indicates that MECI has a pronounced anti-inflammatory effect when it comes to preventing heat-caused protein denaturation.Dose-response curves in Fig 4 show that protein stabilization rises with increasing concentrations of MECI in a pronounced way. This inhibition of protein denaturation suggests that the MECI extract has some bioactive phytoconstituents present--such as phenolic chemicals and flavonoids--which can stabilize proteins and curb inflammation pathways. Taken together, these results demonstrate that the methanolic extract of Combretum indicum has significant anti-inflammatory activity in vitro. This finding supports the use of the material for traditional medicine, and also suggests that it might be a natural source of anti-inflammatory agents.

**Table 4:** Inhibition of protein denaturation by the methanolic extract of *Combretum indicum* (MECI) at different concentrations. The results demonstrate the concentration-dependent anti-inflammatory activity of the extract expressed as percentage inhibition of protein denaturation, along with the calculated IC_50_₅₀ value.

Serial No.	Absorbance of Control	Conc.(µg/ml)	Absorbance of MECI	% inhibition	IC_50_
01	0.144	1000	0.310	95.2778	132.49
02		500	0.264	83.33333	
03		250	0.251	74.30556	
04		125	0.235	63.19444	
05		62.5	0.198	37.5	
06		31.25	0.174	20.83333	

**Fig 4:**
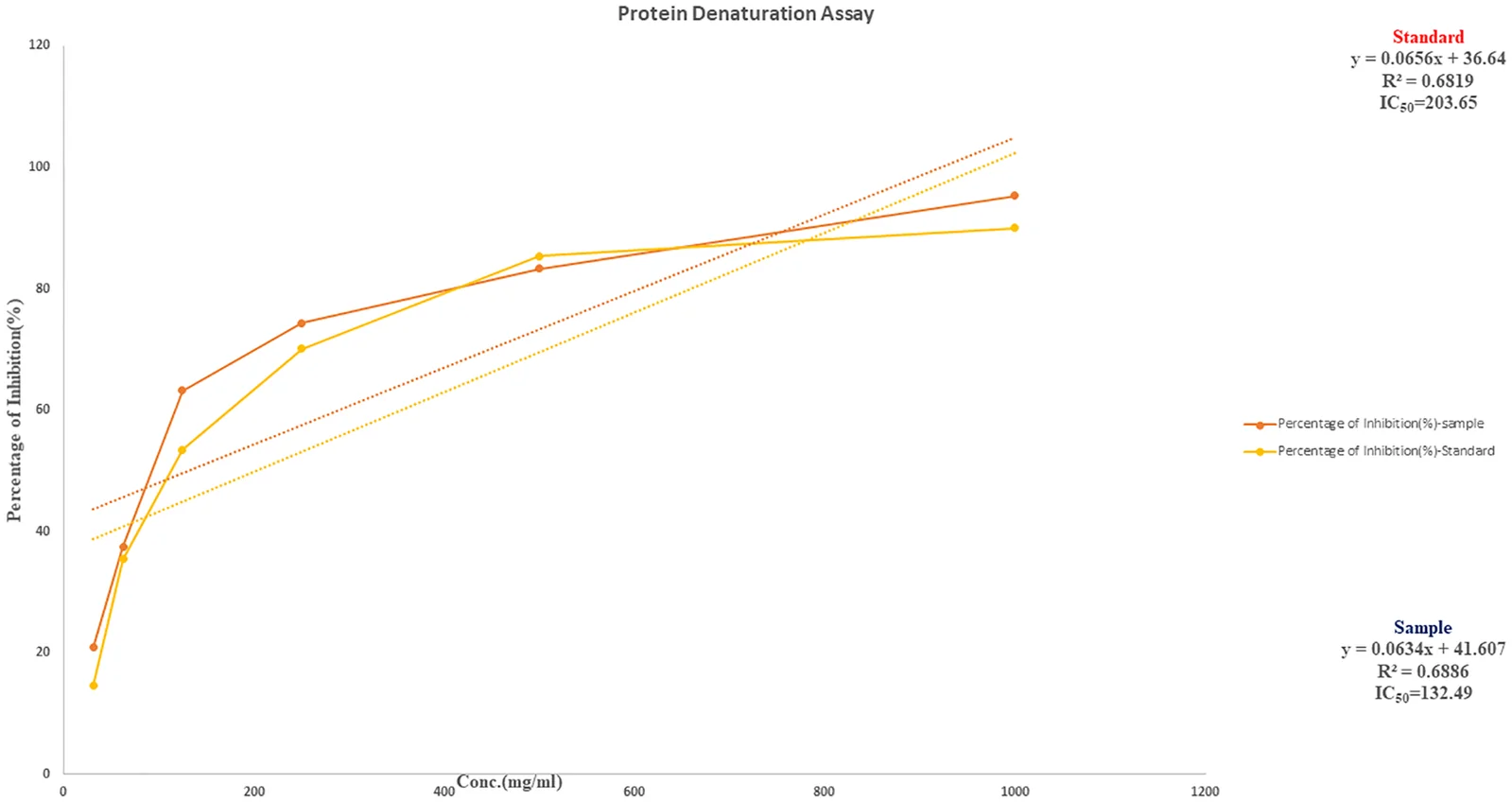
Concentration–response curve illustrating the percentage inhibition of protein denaturation by the methanolic extract of *Combretum indicum* (MECI). The figure highlights the dose-dependent increase in anti-inflammatory activity, with maximum inhibition observed at 1000 µg/mL.

### In vivo carrageenan-induced paw edema anti-inflammatory test

Methanolic extract of *Combretum indicum (MECI)* anti-inflammatory action was examined with the standard Carrageenan induced paw oedema model in animal models, a model of acute inflammation that is well established. Each injection of carrageenan causes the paw edema to develop, promoting a sequential release of inflammatory mediators including histamine, serotonin and prostaglandins. This is illustrated in Table 5. The control group showed a continuous increase in paw thickness during the observation period (0–4 h), thus indicating that we had successfully induced an inflammation. In contrast, animals injected with the anti-inflammatory agent Diclofenac dose (10 mg/kg) showed a substantial drop in any kind of paw edema for the duration of our test process, which by the end 45.45% inhibition.Administering MECI attenuated carrageenan induced paw edema in a dose-dependent manner. At 200 mg/kg MECI, the treated group showed 28.33% inhibition of paw edema compared with the control group. Notably, the 400 mg/kg dose showed a significantly greater degree of inhibition, with around 65% suppression for at least four hours, which practically equaled and even outdone the drug’s activity in this experimental situation.The time course of MECI anti-inflammatory responses showed significant inhibitory effect only in the later phase of inflammation (3–4 h), when prostaglandins and other inflammatory mediators are released. It suggests that the extract might block cyclooxygenase mediated inflammation or stop production of pro-inflammatory mediators. The beneficial impact of MECI on Carrageenan induced paw oedema is so well illustrated in Fig 5. It holds up better than both the control and even more so Diclofenac as time advances. This data overall makes clear that methanolic extract from *Combretum indicum* has demonstrable in vivo anti-inflammatory activity, and could be considered as a natural anti-inflammatory on that basis.

**Table 5:** Effect of the methanolic extract of *Combretum indicum* (MECI) on paw edema at different time intervals in the carrageenan-induced inflammation model. Values are expressed as mean ±  SEM of paw thickness (mm) measured at 0, 1, 2, 3, and 4 hours after carrageenan administration. The standard drug Diclofenac was used as the positive control. The percentage inhibition represents the reduction in paw edema compared with the control group.

Treatment Group (mg/kg)	Paw thickness (Normal condition)	Mean decrease in paw Volume (mm)				% of Decrease paw Volume	
		0H	1H	2H	3H	4H	
Control	1.94 ± 0.01	2.94 ± 0.01	2.03 ± 0.007	1.89 ± .004	2.49 ± 0.007	2.80 ± 0.002	–
Diclofenac(10 mg/kg)	1.90 ± 0.008	3.0 ± 0.01	2.94 ± 0.004	2.64 ± 0.03	2.56 ± 0.001	2.50 ± 0.002	45.45%
MECI-200	1.9 ± 00	3.1 ± 0.03	2.94±	2.84±	2.80±	2.76±	28.33%
MECI-400	2.0 ± 0.04	3.2±	2.86±	2.73±	2.50±	2.42±	65%

**Fig 5:**
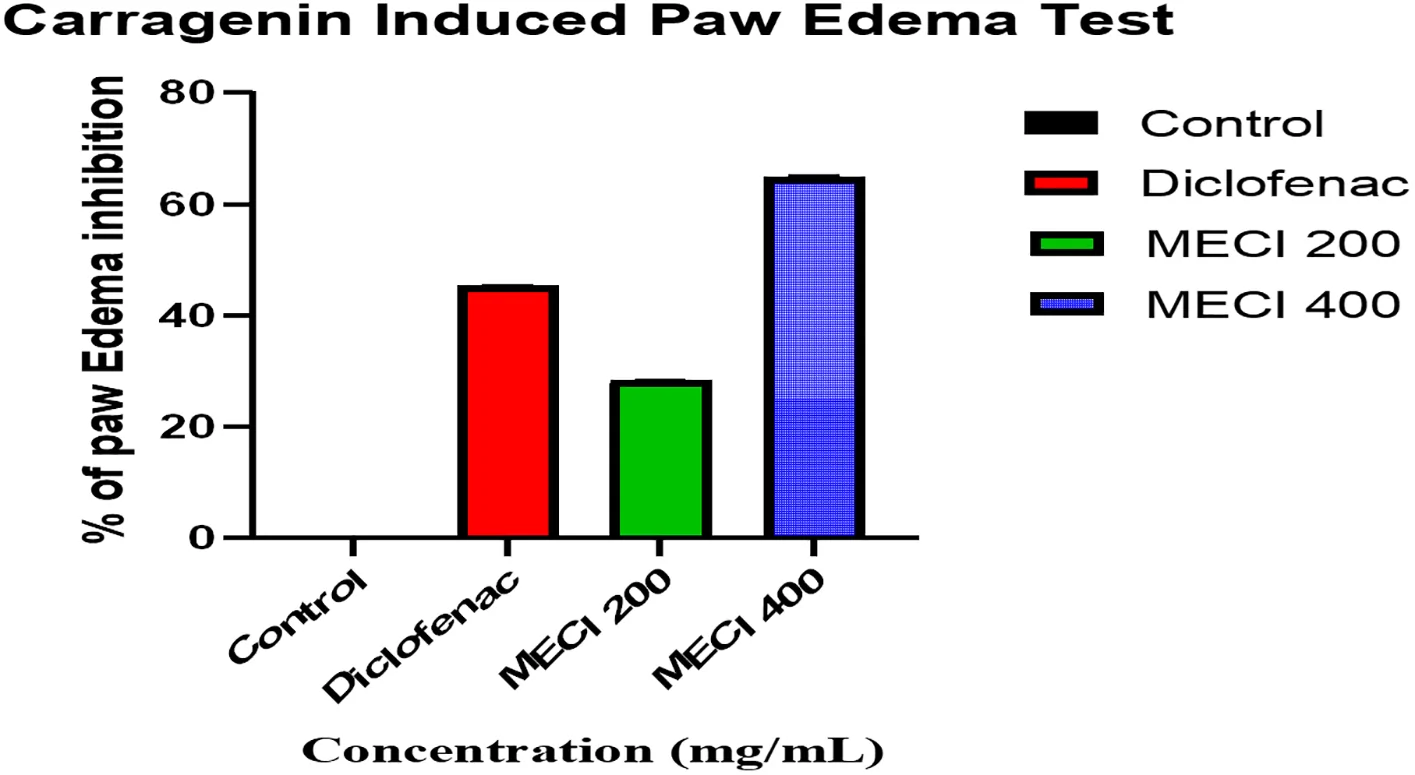
Time-dependent inhibitory effect of the methanolic extract of *Combretum indicum* (MECI) on carrageenan-induced paw edema compared with the control and the standard anti-inflammatory drug Diclofenac. The figure illustrates the dose-dependent reduction of paw swelling, with the highest anti-inflammatory activity observed at 400 mg/kg MECI.

### Evaluation of in vitro and vivo anti-arthritic activity

#### Evaluation of in vitro anti-arthritic activity.

We assessed the in vitro antiarthritic potential of methanolic extract from the leaves of Combretum indicum, using protein denaturation assay as a preliminary screening model for arthritic agents Protein denaturation is a key pathological process related to a variety of inflammatory diseases such as rheumatoid arthritis, where changes in protein structure can lead to autoimmune attack and worsen inflammation [40,63]. At every concentration level examined (31.25–1000 µg/mL), there was a decrease in extent denatured protein with respect to the initial state. As Table 6 below illustrates, inhibition of denaturation activity increased progressively with increasing concentrations. When the extract was administered at its smallest concentration level (31.25 µg/mL), for example, this led to a 52.53% reduction in denaturation. By contrast, stronger inhibition rates of 54.00%, 57.73% and 58.66% were seen at those same concentration levels respectively. When the extract dosage increased still further to 500 µg/mL, the inhibitory effect on denaturation was pushed up to 65.06%. The highest level measured was 85.6% at 1 mg/mL. By conducting the experiment using MECI, an IC_50_ value of 47.15 µg/mL was calculated and thus points to successful inhibition protein denaturation. This inhibitory effect suggests that phytochemical constitution present in *Combretum indicum*, especially phenolic compounds, flavonoids and other antioxidant metabolites, are able to stabilize protein structure and prevent inflammatory protein degradation. As illustrated in Fig 6 (Dose-Response Relationship), with increasing concentrations of MECI, anti-arthritic activity visibly increases. High concentrations of the extract reveal strong inhibiting activity on arthritic development. We can thus infer that *Combretum indicum* is a promising wellspring of bioactive constituents with anti-arthritic effect.In summary, data from this study presents experimental evidence in support of *Combretum indicum’s* therapeutic potential in combating arthritic diseases and its molecules of bioactive constituents as well as first-pass information concerning mechanisms for future studies.

**Table 6:** *In vitro* anti-arthritic activity of the methanolic extract of *Combretum indicum* (MECI) evaluated by the protein denaturation inhibition assay. The data represent the percentage inhibition of protein denaturation at different extract concentrations along with the calculated IC₅₀ value.

Concentration	Absorbance of Sample	Abs. of Product Control	Abs. of Control	% of Inhibition	IC50
31.25	0.437	0.043	0.75	52.53333	47.15
62.5	0.455	0.05	0.75	54	
125	0.492	0.059	0.75	57.73333	
250	0.5	0.06	0.75	58.66667	
500	0.544	0.056	0.75	65.06667	
1000	0.694	0.052	0.75	85.6	

**Fig 6:**
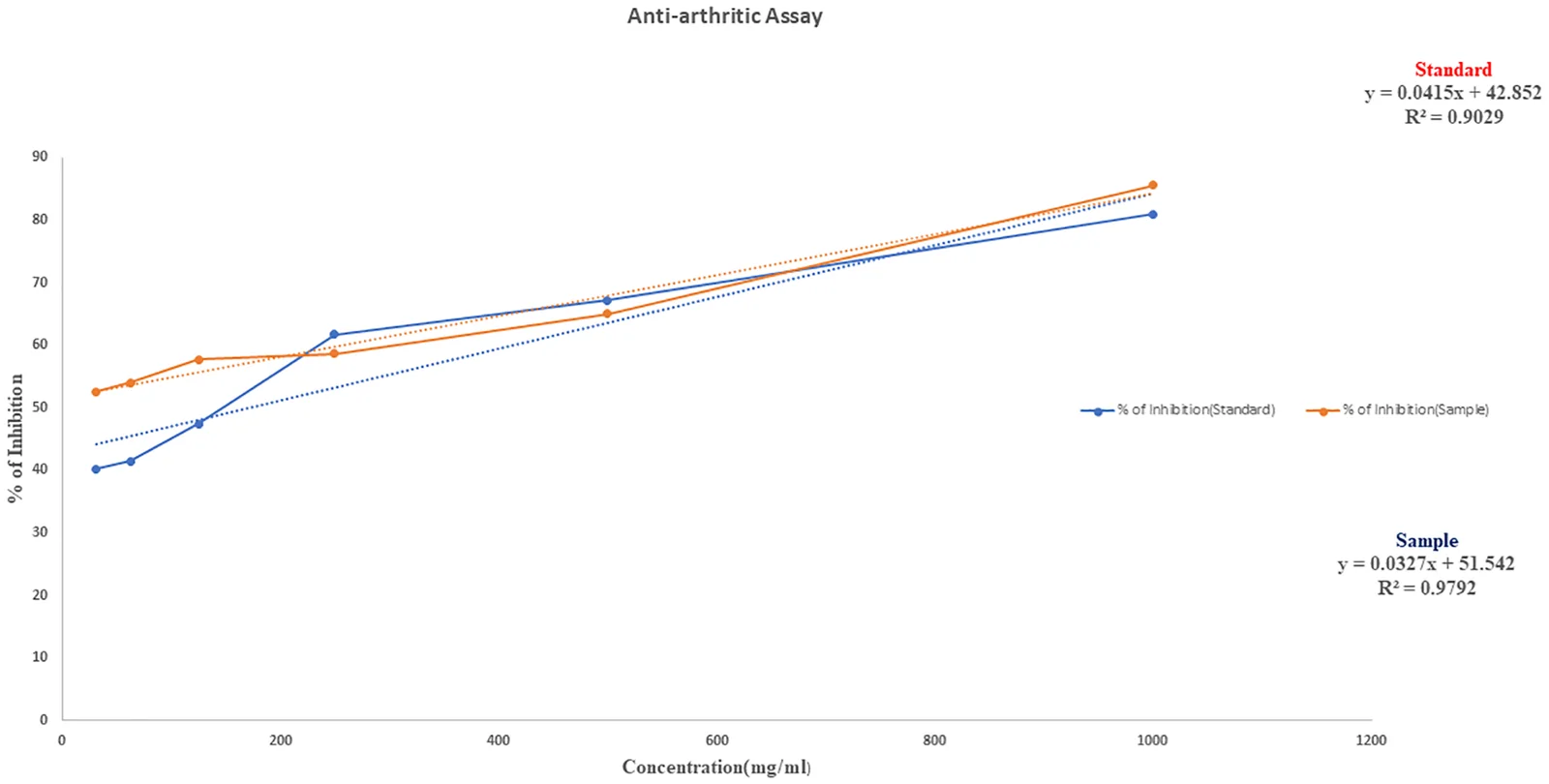
Concentration-dependent inhibition of protein denaturation by the methanolic extract of *Combretum indicum* (MECI). The figure illustrates the dose–response relationship demonstrating enhanced anti-arthritic activity with increasing extract concentration.

### In vivo formaldehyde-induced arthritis test

By employing the formaldehyde-induced arthritis model, a well-established experimental approach for evaluating chronic inflammation response and joint swelling in laboratory animals, we tested to inspect if the methanolic extract of *Combretum indicum* had anti-arthritic activity. This model mimics a several pathologic features of human inflammatory joint disorders like Rheumatoid Arthritis with consistent edema as well as the release of inflammatory mediator.

As illustrated in Table 7, the control group showed a paw diameter of 4.80 ± 0.12 mm, indicating serious inflammatory oedema following formaldehyde administration. In contrast, treatment with the standard anti-arthritic drug Indomathacin (10 mg/kg) significantly reduced paw swelling to 0.90 ± 0.06 mm, equivalent to 81.25% of inhibition of edema.

**Table 7:** Effect of the methanolic extract of *Combretum indicum* on paw diameter in the formaldehyde-induced arthritis model. Values are expressed as mean ±  SEM (n  = 5). The percentage inhibition represents the reduction in paw edema compared with the control group. The standard anti-inflammatory drug Indomethacin (10 mg/kg) was used as the positive control.

Treatment Group	Paw diameter (mm) at 6 h(mean ± SEM (n = 5)	% Edema Inhibition
Control	4.80 ± 0.12	0.00
Indomethacin 10 mg/kg	0.90 ± 0.06	81.25
MECI 200 mg/kg	1.50 ± 0.08	68.75
MECI 400 mg/kg	0.60 ± 0.04	87.50

Animals experiencing this affliction who were treated with the methanolic extract exhibited reduction in paw edema with increase doses. At the 200 mg/kg dose, paw swelling decreased significantly (1.50 ± 0.08 mm) with 68.75% less edema than the control group alone. The 400 mg/kg dosage produced maximum anti-arthritic effect and decreased paw diameter to 0.60 ± 0.04 mm which was 87.50% of inhibition against edema, more so than the standard drug did under these experimental circumstances.

Fig 7 presents in tabular form the striking reduction in paw inflammation resulting from treatment with the methanolic extract of *Combretum indicum*. The pronounced inhibition of formaldehyde-induced arthritis also suggests that the extract achieves its therapeutic effect through inhibition or modulation of inflammatory mediators, lysosomal membrane stabilization and making pro-inflammatory cytokines.

**Fig 7:**
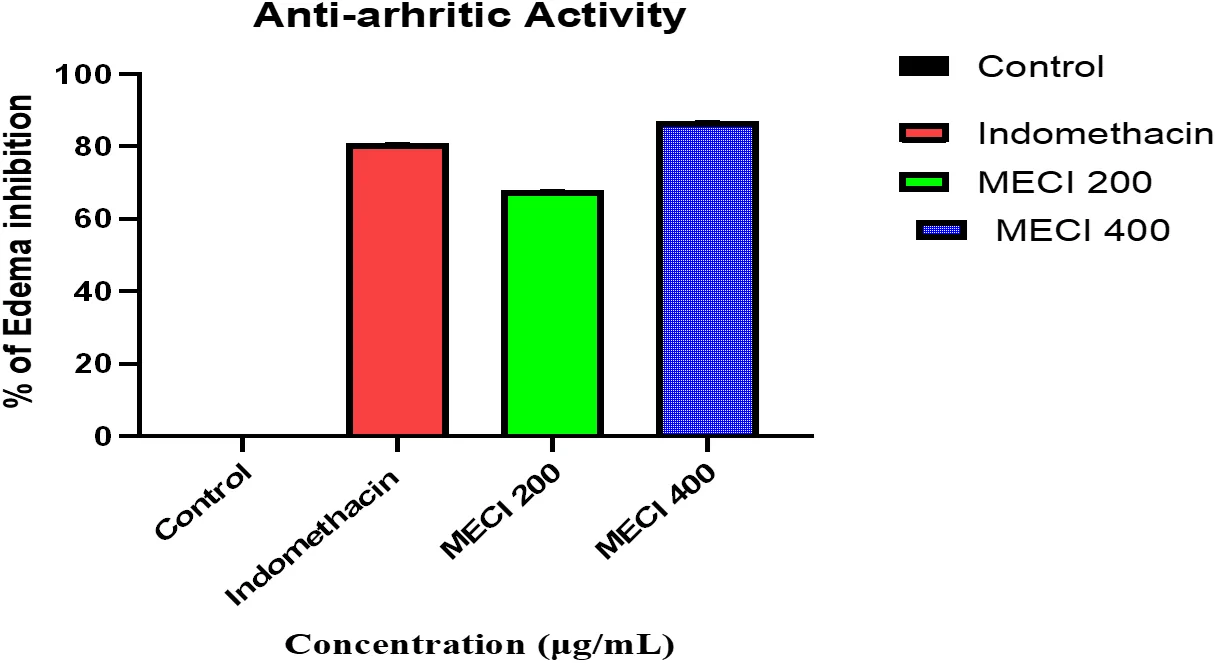
Comparative anti-arthritic effect of the methanolic extract of *Combretum indicum* and the standard drug Indomethacin in the formaldehyde-induced arthritis model. The figure illustrates the dose-dependent reduction of paw edema, with the 400 mg/kg extract showing the highest inhibition of arthritis.

In summary, the findings lead us to suppose that *Combretum indicum* has a potent in vivo anti-arthritic action, which opens up a whole new area for its traditional medicinal application and makes it an important potential source of drug materials to ease chronic inflammatory joint disease.

### Evaluation of in vivo analgesic activity

As shown in Table 8 and Fig 8, the effect of the test compound on writhing behavior in mice is no decrease in activity, suggesting 0% protection As mice in the control group were given a 0.2 ml/mouse intraperitoneal injection of saline, they scored no decrease at all in the number of writhes. This is the baseline behavior or 0% protection.

**Table 8:** Effect of test compound on writhing behavior in mice. Values are expressed as mean ±  SEM (n  =  6). % Protection calculated relative to saline control. Statistical significance: p<0.01 () and p <  0. 001 (***) versus control.

Group	Dose	No of Writhes	% of Protection
Saline (Control)	0.2ml/mouse,ip	0	0
Duclofenac (Standard)	20mg/kg, ip	8.54 ± 0.67***	78.05%
MECI-200 mg	200mg/kg, sc	10.56 ± 0.13**	60.12%
MECI-400 mg	400mg/kg,sc	9.43 ± 0.43**	70.15%

**Fig 8:**
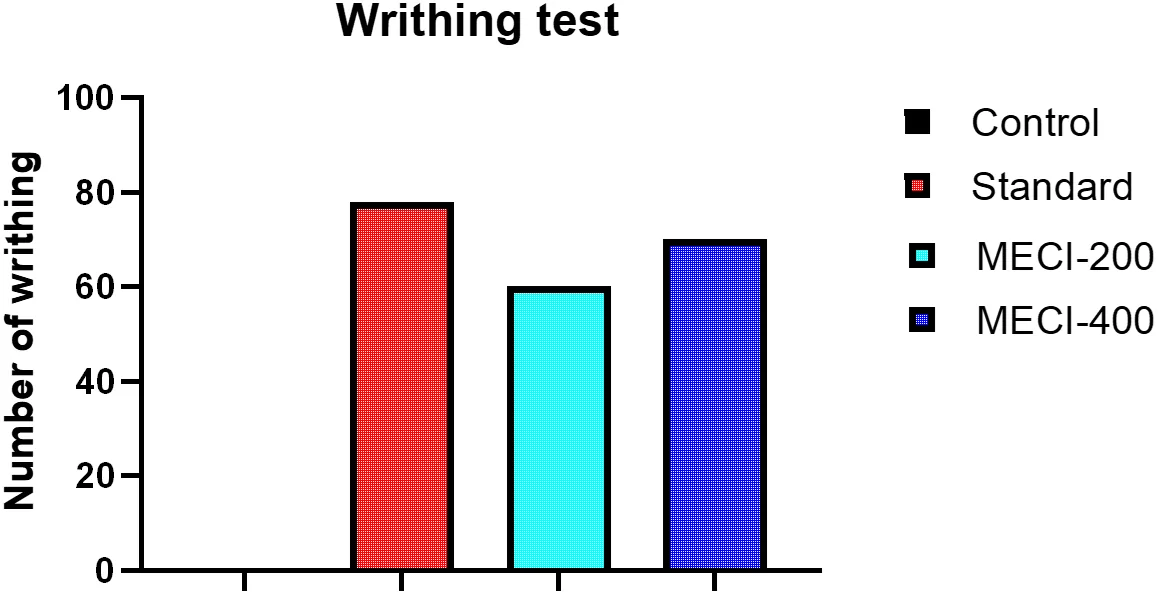
Protective effect of test compound on writhing behavior in mice. Data represent mean ± SEM (n = 6). ** indicates p < 0.01, *** indicates p < 0.001 compared to saline control.

The standard drug, Diclofenac (20 mg/kg, intraperitoneally), showed a significant decrease in the number of writhes. In the control group, this resulted in 8.54 ± 0.67 writhes, accounting for 78.05% protection (P < 0.001). It points to strong anti-inflammatory and protective power of the drug in this experimental model.

For the test compound, either dose caused significant reduction in writhing behavior. When the points mean was 200 mg/kg (subcutaneous), number of writhes scored 10.56 ± 0.13 with this corresponding to 60.12% protection (p < 0.01). With the higher dose of 400 mg/kg (subcutaneous) scoring was 9.43 ± 0.43 writes and 70.15% protection (p < 0.01). Both of these findings make it clear there is a dose-dependent protective effect by test compound approaching that achieved with standard drug.

### Evaluation of in vitro cytotoxic activity (Brine Shrimp Lethality Assay)

The cytotoxic potential of the methanolic extract of *Combretum indicum* was assessed using the brine shrimp lethality assay, a standard preliminary test for identifying biologically active compounds with possible anticancer, pesticidal, or pharmacological properties. This assay measures the effect of a substance on the survival of *Artemia salina nauplii*, providing an indication of general cytotoxicity.

The extract showed a clear concentration-dependent effect on brine shrimp mortality. At the highest concentrations (500 and 1000 µg/mL), 100% of the nauplii were killed. Intermediate concentrations also caused significant lethality, with 90% mortality at 250 µg/mL and 80% at 62.5–125 µg/mL, while the lowest concentration (31.25 µg/mL) produced 30% mortality. The calculated LC₅₀ value was 30.19 µg/mL, highlighting the extract’s strong cytotoxic potential (Table 9 and Fig 9).

**Fig 9:**
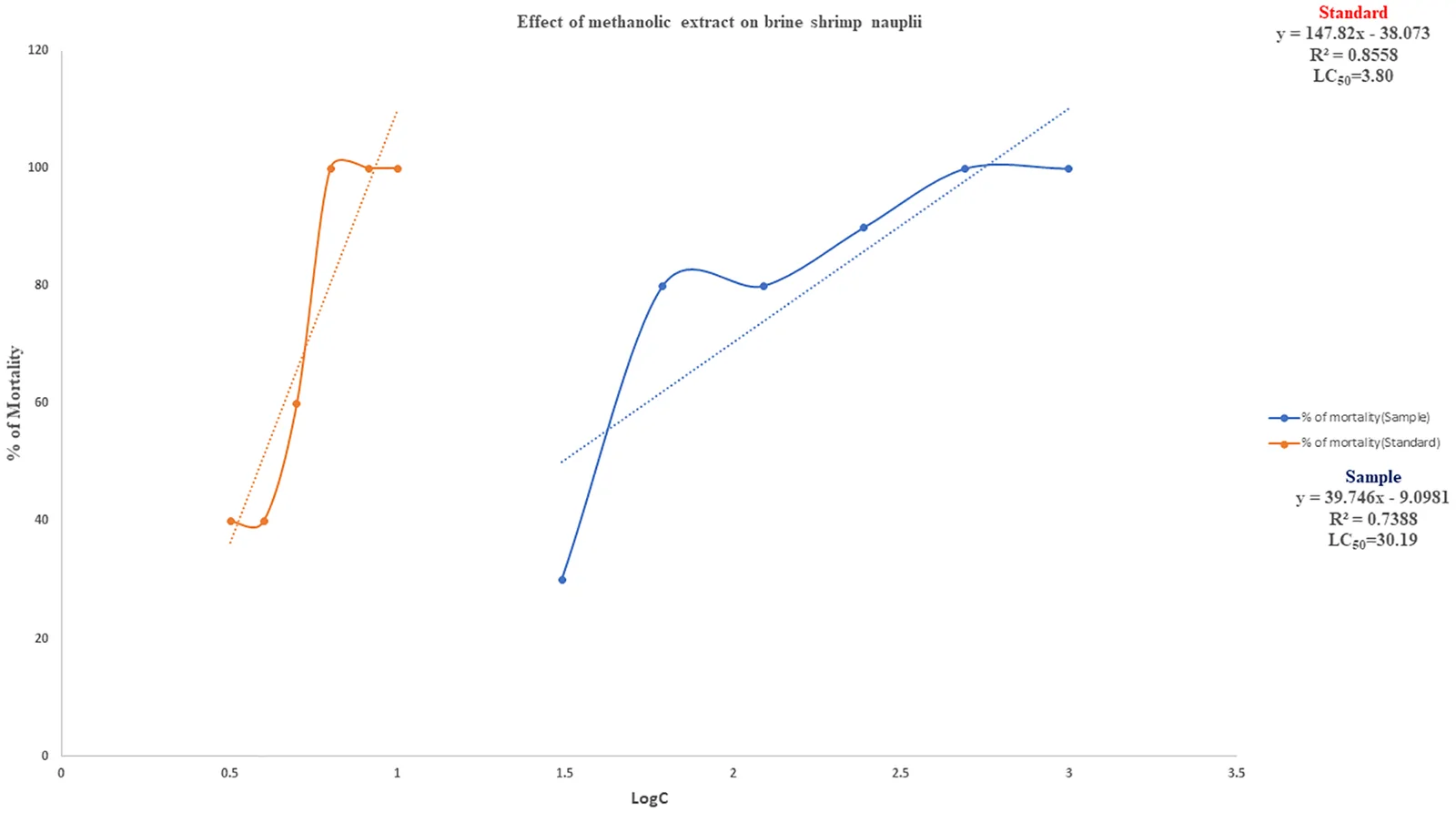
Dose–response relationship showing the percentage mortality of *Artemia salina nauplii* exposed to different concentrations of the methanolic extract of *Combretum indicum.* The graph illustrates the concentration-dependent cytotoxic effect, with higher mortality observed at increased extract concentrations.

This activity is likely due to bioactive phytochemicals such as alkaloids, flavonoids, and phenolic compounds. The dose–response relationship confirms that *C. indicum* contains potent constituents capable of cytotoxic effects, supporting its potential medicinal and drug discovery applications.

**Table 9:** *In vitro* cytotoxic activity of the methanolic extract of *Combretum indicum* evaluated by the brine shrimp lethality assay using *Artemia salina iinaupl*. The table presents the number of alive and dead nauplii at different extract concentrations along with the calculated percentage mortality and LC_50_ value.

Concentration(mg/ml)	LogC	N. of naupii	Alive	Dead	% of mortality	LC_50_(µg/ml)
1000	3	10	0	10	100	30.19
500	2.69	10	0	10	100	
250	2.39	10	1	9	90	
125	2.09	10	2	8	80	
62.5	1.79	10	2	8	80	
31.25	1.49	10	3	7	30	

### Evaluation of anti-helminthic activity

The anthelmintic potential of the methanolic extract of *Combretum indicum* (MECI) was investigated by measuring the paralysis time and death time of helminths exposed to different concentrations of the extract. Anthelmintic activity is typically assessed by the ability of a compound to induce neuromuscular paralysis followed by death of parasitic worms, thereby interrupting their physiological functions. The experimental results revealed a concentration-dependent anthelmintic effect of MECI. As summarized in Table 10, increasing concentrations of the extract significantly reduced both paralysis time and death time of the worms. At the lowest tested concentration (50 mg/mL), MECI produced paralysis and death at 42.6 ± 0.89 min and 74 ± 2.12 min, respectively. Increasing the concentration to 100 mg/mL markedly accelerated the anthelmintic effect, resulting in paralysis and death at 8.8 ± 1.92 min and 52.2 ± 0.83 min, respectively. A further enhancement of activity was observed at 150 mg/mL, where worms were paralyzed within 5 ± 0.70 min and died after 17.6 ± 1.14 min. The highest concentration (200 mg/mL) exhibited the most potent effect, causing paralysis within 3.6 ± 0.89 min and death within 8 ± 0.70 min, indicating strong anthelmintic activity of the extract. For comparison, the standard anthelmintic drug Albendazole produced paralysis and death at 23.2 ± 3.63 min and 54.2 ± 1.30 min, respectively. Notably, the higher concentrations of MECI demonstrated faster anthelmintic action than the standard drug, suggesting the presence of potent bioactive phytochemicals capable of interfering with the neuromuscular system and metabolic processes of parasitic worms. The graphical representation in Fig 10 clearly illustrates the concentration-dependent decrease in paralysis and death times with increasing extract concentrations. These findings indicate that *Combretum indicum* possesses significant anthelmintic properties, which may be attributed to the presence of secondary metabolites such as tannins, flavonoids, and alkaloids known for their antiparasitic activity. Overall, the results demonstrate that the methanolic extract of *Combretum indicum* exhibits pronounced anthelmintic activity, supporting its potential application as a natural antiparasitic agent and warranting further investigation into its active constituents and mechanism of action.

**Table 10:** Anthelmintic activity of the methanolic extract of *Combretum indicum* (MECI) showing mean paralysis time and death time (mean ±  SEM) of helminths at different extract concentrations compared with the standard drug Albendazole.

Concentration(mg/ml)	MEAN±SEM(Death time)	MEAN±SEM(Paralysis time)
MECI-50	74 ± 2.12	42.6 ± 0.89
MECI-100	52.2 ± 0.83	8.8 ± 1.92
MECI-150	17.6 ± 1.14	5 ± 0.70
MECI-200	8 ± 0.70	3.6 ± 0.89
Standard	54.2 ± 1.30	23.2 ± 3.63
Control	0	0

**Fig 10:**
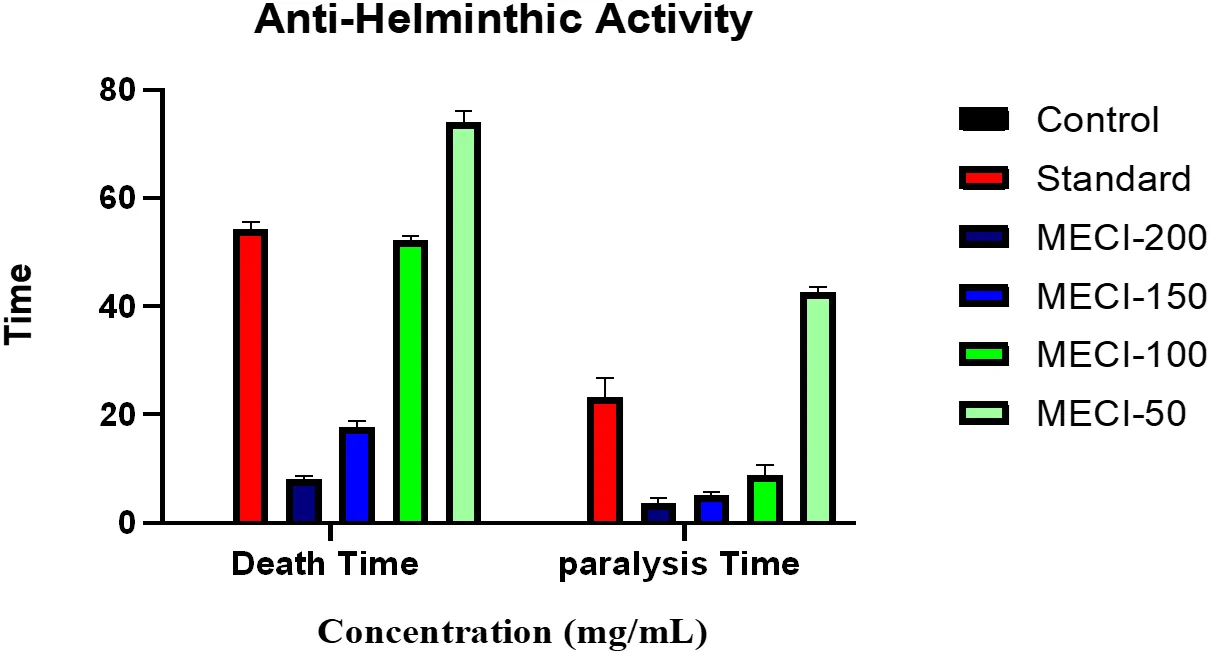
Comparative anthelmintic activity of the methanolic extract of *Combretum indicum* at different concentrations and the standard drug Albendazole. The bar graph illustrates the concentration-dependent reduction in paralysis and death time of helminths, indicating strong anthelmintic potential of the extract.

#### *In silico*.

BOILED-Egg Model for Prediction of Gastrointestinal Absorption and Blood–Brain Barrier Permeability:

The Brain Or IntestinaL Estimated (BOILED-Egg) permeation method is an accurate predictive model that estimates the lipophilicity and polarity of small molecules, ranging from early screening of chemical libraries in drug discovery to the evaluation of potential drug candidates for further development. This method simultaneously predicts permeability in both the brain and gut using the same physicochemical descriptors. Therefore, this prediction was performed for the selected compounds and miltefosine using the swissADME tool, as depicted in Fig 11.

**Fig 11:**
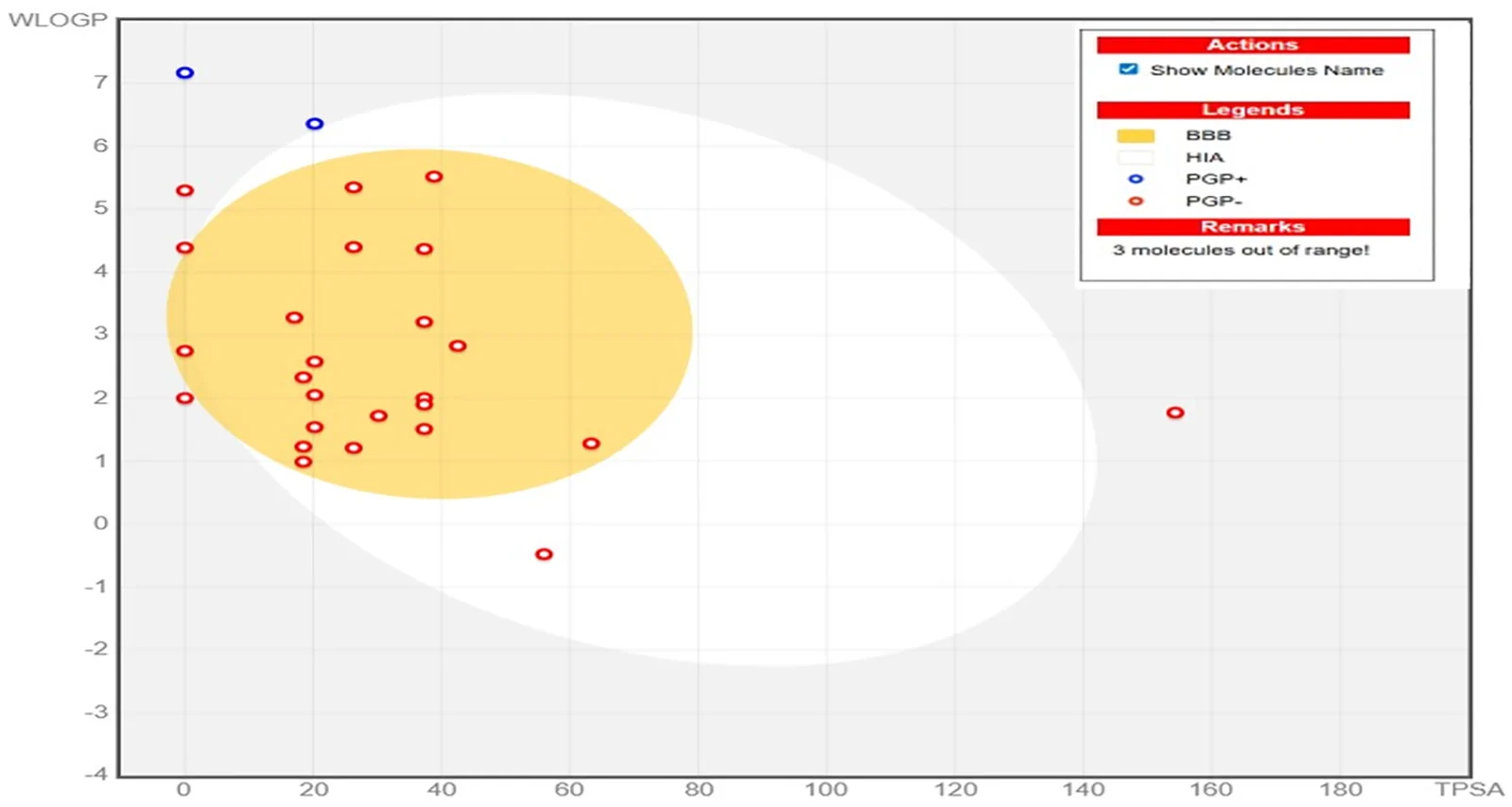
BOILED-Egg graph of *Combretum indicum* representing in silico prediction of human intestinal absorption (HIA) and blood–brain barrier (BBB) penetration. The white region is for a high probability of passive absorption by the gastrointestinal tract, and the yellow region is for a high probability of brain penetration. In addition, the points are colored in blue if predicted as actively effluxed by P-gp (PGP+) and in red if predicted as non-substrate of P-gp (PGP−).

### In silico evaluation of antioxidant activity

Molecular docking analysis was conducted to evaluate the antioxidant potential of the chosen compounds against target protein1DNU. The docking scores of the tested compounds compared with the reference antioxidant compound are shown in Table 11. Among the compounds tested, the highest affinity binding of any molecule with a docking score of −9.8 kcal/mol, indicating strong interaction with the active site of the protein, was seen for methyl tetradeca-12,13-dienoate. This binding energy was significantly better than that of the reference antioxidant compound Ascorbic acid, which registered a docking score of −9.0 kcal/mol.Similarly, tetrazolo[1,5-b]pyridazine exhibited strong interaction with the target protein angiotensin, scoring a docking value as low as −9.5 kcal/mol--though not so great as dibutan-2-yl sulfite at −9.1 kcal/mol. These results suggest the tested compounds show similar or even greater binding capacity compared with standard antioxidants. Table 11 summarizes detailed information on docking scores for all of these compounds. Closer analysis of amino acid residue-ligand interactions with the protein revealed that the selected compounds formed several stabilizing interactions at key sites within the protein’s pocket. The specific residues of binding ligands with protein are shown in Table 12. These interactions are primarily hydrogen bonding, hydrophobic contacts, and van der Waals forces. These collectively contribute to the stability of the ligand–protein complex. The Fig 12 shows the three-dimensional binding pose of the top-scoring compounds within the active site of protein 1DNU, emphasizing how they are oriented and what interactions they have with surrounding amino acid residues. Methyl tetradeca-12,13-dienoate and tetrazolo[1,5-b]pyridazine have promising antioxidant binding potentials, on the basis of docking results. This suggests that these could be candidates for further pharmacological and molecular dynamics studies.

**Table 11:** Docking scores of the investigated compounds and standard reference drugs against seven pharmacological target proteins: Antioxidant (1DNU), thrombolytic (2HLO), anti-inflammatory (1CX2), anti-arthritic (6RMJ), analgesic (5IKR), cytotoxic (1NME), and anthelmintic (1SA0). The table presents the binding energies (kcal/mol) indicating the relative binding affinities of each ligand within the active site of the respective protein.

Compound Name	Anti-oxidant	Thrombolytic	Anti-inflammatory	Anti-arthritic	Analgesic	Cytotoxic	Anthelmintics
	PDB ID:(1DNU)	PDB ID:(2HLO)	PDB ID:(1CX2)	PDB ID:(6RMJ)	PDB ID:(5IKR)	PDB ID:(1NME)	PDB ID:(1SA0)
Methyl tetradeca-12,13-dienoate	−9.8						
Tetrazolo[1,5-b]pyridazine	−9.5					−8.3	
Dibutan-2-yl sulfite	−9.1						
5-Nitro-2-aminobenzophenone thiosemicarbazone		−9.3					−10.6
3-propylcyclohexene			−9.3	−10.6			
12-bromododecanoic acid			−9.9				
Decanoic acid				−9.4			
2-butylthiolane 1,1-dioxide				−10.5			
3,11-dimethylspiro[5.5]undecane			−9.6		−8.7		
2,3,4-trimethylpentanoic acid						−8.5	
Methyl 10,13-dimethyltetradecanoate						−8.0	
Ascorbic Acid	−9.0						
Streptokinase		−9.0					
Diclofenac			−8.5		−8.3		
Naproxen				−8.3			
Methotrexate						−7.8	
Albendazole							−9.8

**Table 12:** Amino acid residues involved in ligand–protein interactions for all seven pharmacological targets. The table lists hydrogen bonds, hydrophobic contacts, and other key interactions between each investigated compound and the residues located within the active binding pockets of antioxidant (1DNU), thrombolytic (2HLO), anti-inflammatory (1CX2), anti-arthritic (6RMJ), analgesic (5IKR), cytotoxic (1NME), and anthelmintic (1SA0) proteins.

Activity	Compound Name	Binding Affinity (PyRx, kcal/mol)	Types of Bonds	Amino Acid Residues
Anti-oxidant(PDB ID:1DNU)	Methyl tetradeca-13,13-dienoate	−9.8	Vander waals	GLY A:110, GLU A:492, ASN A:125.
			C-H Bond	ARG A:109, PHE A:112.
			Alkyl	ALA A:111, TRP A:128.
			Pi-Alkyl	TYR A:124, HIS A:488.
	Tetrazolo[1,5-b] pyridazine	−9.5	Vander waals	ASP A:132, THR A:204, THR A:205.
			C-H Bond	GLU A:492
			Pi-Alkyl	ARG A:129
			Conventional Hydrogen Bond	HIS A:488, ASN A:125.
	Dibutan-2-yl sulfite	−9.1	Vander waals	LEU A:176, TYR A:175, LUS A:357, SER A:334.
			Pi-Alkyl	ARG A:172
			Conventional Hydrogen Bond	GLU A:329, ASP A:328
	Ascorbic Acid	−9.0	Vander waals	PHE A:352, GLN A:215.
			C-H Bond	PHE A:208
			Pi-Alkyl	ILE A:335.
			Alkyl	ILE A;325, LEU A:337, ILE A;180, TYR A:444.
Thrombolytic Activity(PDB ID:2HLO)	5-Nitro-2-aminobenzophenone thiosemicarbazone	−9.3	Vander waals	PRO C:243, MET C:264, ARG C’;275
			Pi-Alkyl	ALA C:279, LYS C:266
			Conventional Hydrogen Bond	ALA C:241, LYS C:302
	Strptokinase	−9.0	Vander waals	THR A:238, PHE F:303, MET C;264, THR C:277.
			Alkyl	LYS F:302, LYS C:266
			Conventional Hydrogen Bond	ASP F:291, SER F:306, ASN F:308
Anti-inflammatory(1CX2)	12-bromododecanoic acid	−9.9	Vander waals	PRO A:547, GLU A;553, MET B:48, THR B;60, ASP B:58, SER A:548
			Alkyl	LYS B:56
	Decanoic acid	−9.6	Vander waals	PRO B:474, ILE B:498, LYS B:473, SER B:477
			C-H Bond	GLU B:480
			Conventional Hydrogen Bond	GLU B:510
			Co-valent	PRO B:474, THR B:476
			Pi-Alkyl	LEU B:481
	3-propylcyclohexene	9.3	Vander waals	PRO A:40,SER A:38.
			Co-valent	ASN A:68
			Conventional Hydrogen Bond	GLU A:67,ASN A:68.
	Diclofenac	−8.5	Vander waals	SER A:38, PRO A;40, TYR A;55.
			Co-valent	ASN A:68.
			Carbon-Hydrogen Bond	GLU A:67, ASN A:68
Anti-arthritic (PDB ID:6RMJ)	3-propylcyclohexene	10.6	Vander waals	LYS C:98, LYS B:98, GLN A:68.
			Alkyl	PRO A:117.
	2-butylthiolane 1,1-dioxide	10.5	Vander waals	PRO B:100, CYS B:101, GLN B:102, PRO A:100.
			Alkyl	ARG B:103, ARG C:103.
			Conventional Hydrogen Bond	GLN A:102.
	Decanoic acid	−9.4	Vander waals	ASN B:39, ALA B;14, VAL B:41
			C-H Bond	ILE B:154, ALA B:156, LEU B:37.
			Conventional Hydrogen Bond	ALA B:38.
			Co-valent	ALA B:14, PRO B:12.
			Alkyl	ILE B:155, LEU A:55.
	Diclofenac	−8.3	Vander waals	ALA B:145, ASP B:143, TYR B:141, GLU B:23.
			C-H Bond	PHE B:144
			Conventional Hydrogen Bond	GLY B:24, LEU B:142, ASP B:140.
			Pi-Sigma	PRO B:20
Analgesic(PDB ID:5IKR)	3,11-dimethylspiro[5.5]undecane	−8.7	Vander waals	LEU A;384, PHE A:381, MET A:522, PHE A:518, GLY A:526.
			Alkyl	TYR A:385, TRP A:387, VALA:349, LEU A:352.
			Pi-Alkyl	ALA A:527, VAL A:523
	Diclofenac	−8.3	Vander waals	TYR A:387, TRO A:387, MET A:522, LEU A:359.
			Conventional Hydrogen Bond	TYR A:355
			Pi-Alkyl	ALA A:527, VAL A:349.
			Pi-Sigma	LEU A:352. VAL A:523.
Cytotoxic(PBD ID:1NME)	Tetrazolo[1,5-b]pyridazine	−8.5	Vander waals	ARG A:164, VAL B:266, MET B:268, GLY A:125.
			Conventional Hydrogen Bond	TYR B:195.
			Pi-Alkyl	VAL B:266
	2,3,4-trimethylpentanoic acid	−8.3	Vander waals	LEU A:136, PRO B:201, MET B:268, GLY A:125.
			Alkyl	TYR B:195, TYR B:197.
			Pi-Alkyl	VAL B:266
			C-H Bond	LYS A:137.
	Methyl 10,13-dimethyltetradecanoate	−8.0	Vander waals	LYS A:137, ARG A:164,THR A:140.
			Alkyl	TYR B:195
			Pi-Alkyl	LEU A:136.
			C-H Bond	TYR A:125
	Methotrexate	−7.8	Conventional Hydrogen Bond	GLN A:161, CYS A:163, GLY A:122,SER A:205.
			Co-valent	CYS A:163
			Vander waals	ALA A:162, SER A:120,TYR B:204, TRP B:206
Anti-helminthic(PDB ID:1SAO)	5-Nitro-2-aminobenzophenone thiosemicarbazone	−10.6	Vander waals	SER B:140, GLY B:144, GLY B:10, GLY B:13, GLY B:143, GLY B:142.
			Conventional Hydrogen Bond	GLN B:11, SER B:147, GLY B:146, ASN B:101.
			Pi-Sigma	CYS B:12.
			Pi-Pi Stacked	TYR B:224.
	Albendazole	−9.8	Vander waals	GLU B:71, GLN B:11, THR B:145, GLY B:144, SER B:147, GLY B:143
			Conventional Hydrogen Bond	GLY B:142.
			Pi-Sigma	CYS B:12
			C-H Bond	ASN B:101, GLY B:10.
			Pi-Anion	ASP B:179

**Fig 12:**
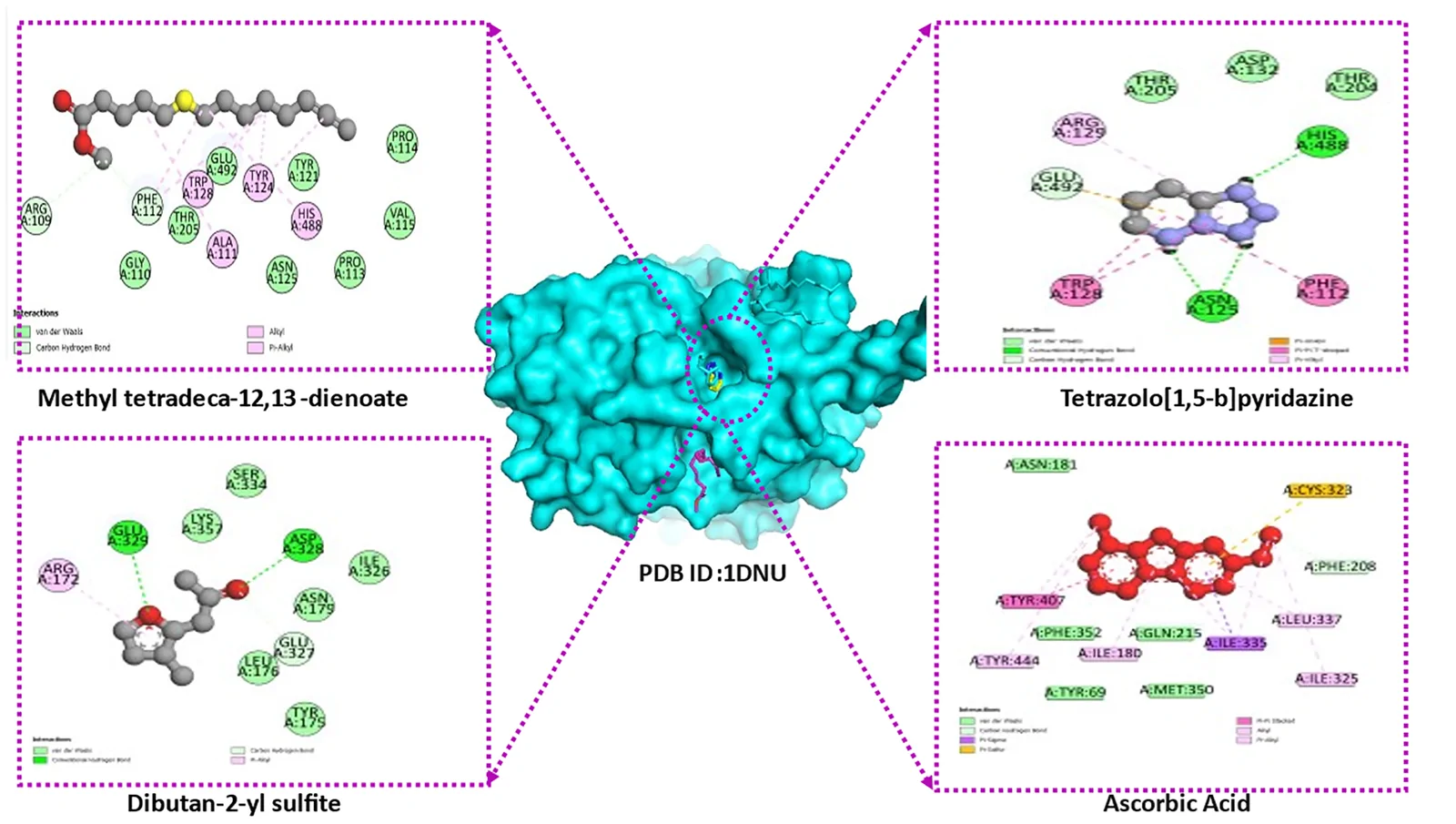
Three-dimensional docking pose of the top-ranked compounds in the active site of the antioxidant target protein 1DNU, illustrating key ligand–protein interactions.

### Molecular docking analysis for thrombolytic activity

To evaluate the activity of the selected compound as a thrombolytic reagent, molecular docking analysis targeting 2HLO was undertaken. The Table 11 is a summary of the docking scores (in Table 11), both of this compound and that of a known agent for dissolving blood clots. This shows that the compound was docked into the active binding pocket of the target protein with a binding energy of −9.3 kcal/mol. Of particular note, this was actually slightly higher than the binding energy of standard thrombolytic drug Streptokinase, which achieved a docking score of −9.0 kcal/mol. The interacting amino acid residues that make up ligand- protein are listed in detail in Table 12. An analysis of the interactions revealed that the ligand lays down this conformation in the hole and through both regions where main chain atoms exist; it maintains stability at that position because its act on protein for binding favorable interactions see an increase. The position of 5-Nitro-2-Aminobenzophenone Thiosemicarbazone in the binding site on the protein is shown in three dimensions in Fig 13, which also illustrates how in front view (i.e., away from viewer) ligand lies; this gives an idea of its relationships with surrounding amino acids. Finally, the results of docking calculations imply that circumstances are such that 5-Nitro-2-aminobenzophenone thiosemicarbazone appears to have binding strength equal to, if not slightly better than, that of streptokinase. Therefore, it shows potential as an attractive candidate for further detailed investigation into thrombolytic drug development work.

**Fig 13:**
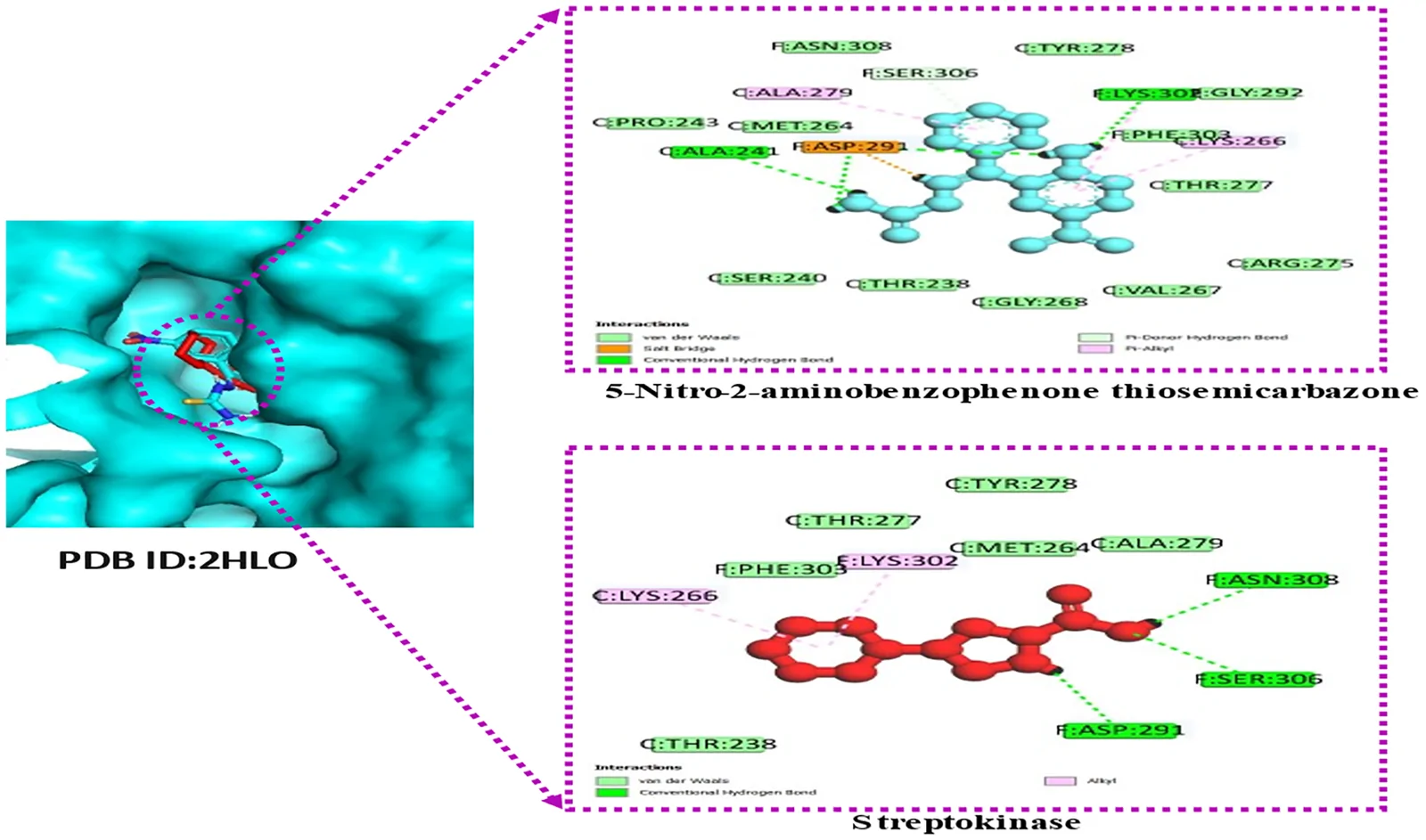
Three-dimensional binding pose of the top-scoring ligand in the active binding pocket of the thrombolytic target protein 2HLO.

### Molecular docking analysis for anti-inflammatory activity

In this case molecular docking has shown that the selected compounds have many anti-inflammatory potential. The molecular docking binding energies of the compounds under test and the standard anti-inflammatory drug are listed together in Table 11 for comparison.How did the prepared compounds compare with respect to how well a known standard might work? 12-Bromo-dodecanoic acid showed a docking score of −9.9 kcal/mol, indicative of an especially strong binding within the binding pocket of target protein. This value is markedly larger than that obtained for Diclofenac, which gave a docking score of −8.5 kcal/mol.What binding energies were shown for 3,11-dimethylspiro[5.5]undecane and 3-propylcyclohexene? They received scores of −9.6 and −9.3 kcal/mol, respecitvely. This implies that they, too, managed to dock well into the active site of protein targets. A detailed analysis of the interactions involved showed that the ligand formed several types of stable contact with key amino acid residues in the binding pocket of the protein. The specific amino acids participating in these interactions are listed in Table 12. These ligand-protein interactions include hydrogen bonding, hydro-phobic effects, van der Waals forces and so forth, collectively contributing to the stability of the system. The three-dimensional binding conformations of this pair of top-scoring compounds in the active binding pocket of the protein are illustrated in Fig 14 to the right and below, showing how they orient themselves with respect to surrounding amino acids and give rise to intermolecular interactions. The results of these docking experiments show that 12-bromododecanoic acid, 3,11-dimethylspiro[5.5]undecane and 3-propylcyclohexene give better binding energies than diclofenac, a common anti-inflammatory drug. This raises the question of whether these compounds deserve to be considered for further investigation on in vitro and in vivo models.

**Fig 14:**
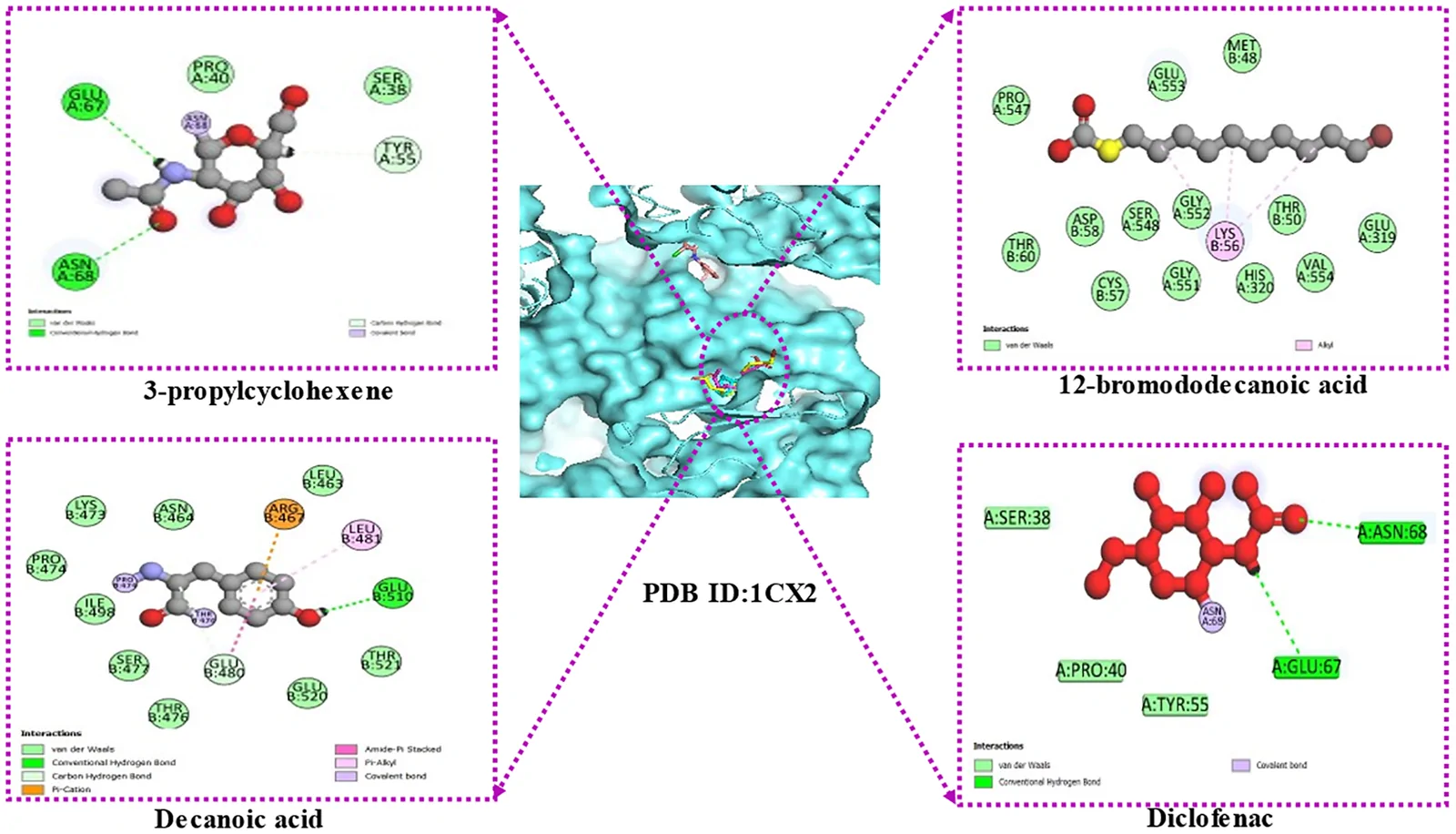
Three-dimensional docking pose of the top-scoring compounds within the active site of the anti-inflammatory target protein 1CX2, highlighting key ligand–protein interactions.

### Molecular docking analysis forctivityarthritic anti-a

Molecular docking analysis is sought after, the anti-arthritic potential of the compounds just isolated. The results of docking analysis calculated for select samples and the reference anti-arthritic treatment—it’s all here in Table 11. Among those drugs examined in this experiment, 3-propylcyclohexene was shown to possess the highest binding power, with a docking score of −10.6 kcal mol − 1 indicating very intimate contact between ligand and site. Similarly, 2-butylthiolane 1,1-dioxide also showed a binding power of −10.5 kcal mol − 1, this number can be seen to be in line with the degree of ligand–active site protein interaction.In addition to these compounds, the compound decanoic acid was brought into focus with its significant binding strength and a docking score on the other hand of −9.4 kcal mol − 1. By comparison, the reference anti-arthritic drug naproxen exhibited a binding affinity of −8.3 kcal/mol. Most of the selected phytochemicals demonstrated favorable binding interactions with key amino acid residues within the active site of the target protein, suggesting their potential as anti-arthritic agents. Details are given in Table 12 below about exactly which residues participate in interaction where the compound stabilizes against protein. These interactions are all hydrophobic contacts and hydrogen-bonding or van der Waals interactions which together keep this complex stable enough for it to function correctly. This shows the top-scoring compound of molecular docking linked with 6RMJ in 3D docked within its proteinase active site, and so also graphically illustrates why these numbers appear to be what they are- if we reposition our minds just a little bit from looking at that whole set of detailed data in Fig 15 to now focus on the spatial configuration of relationships for some two or three dozen atoms which constitute a ligand and residue environment acceptance (or not) by neighboring nitrogen atom is either controlled caudally or dorsally as well as laterally.The outcome of the docking results demonstrates that both 3-propylcyclohexene and,2-butylthiolane 1,1-dioxide were able to achieve substantially higher binding strength compared with Naproxen as a reference drug, It’s possible that these compounds will offer something worthwhile for future investigation (Fig 15).

**Fig 15:**
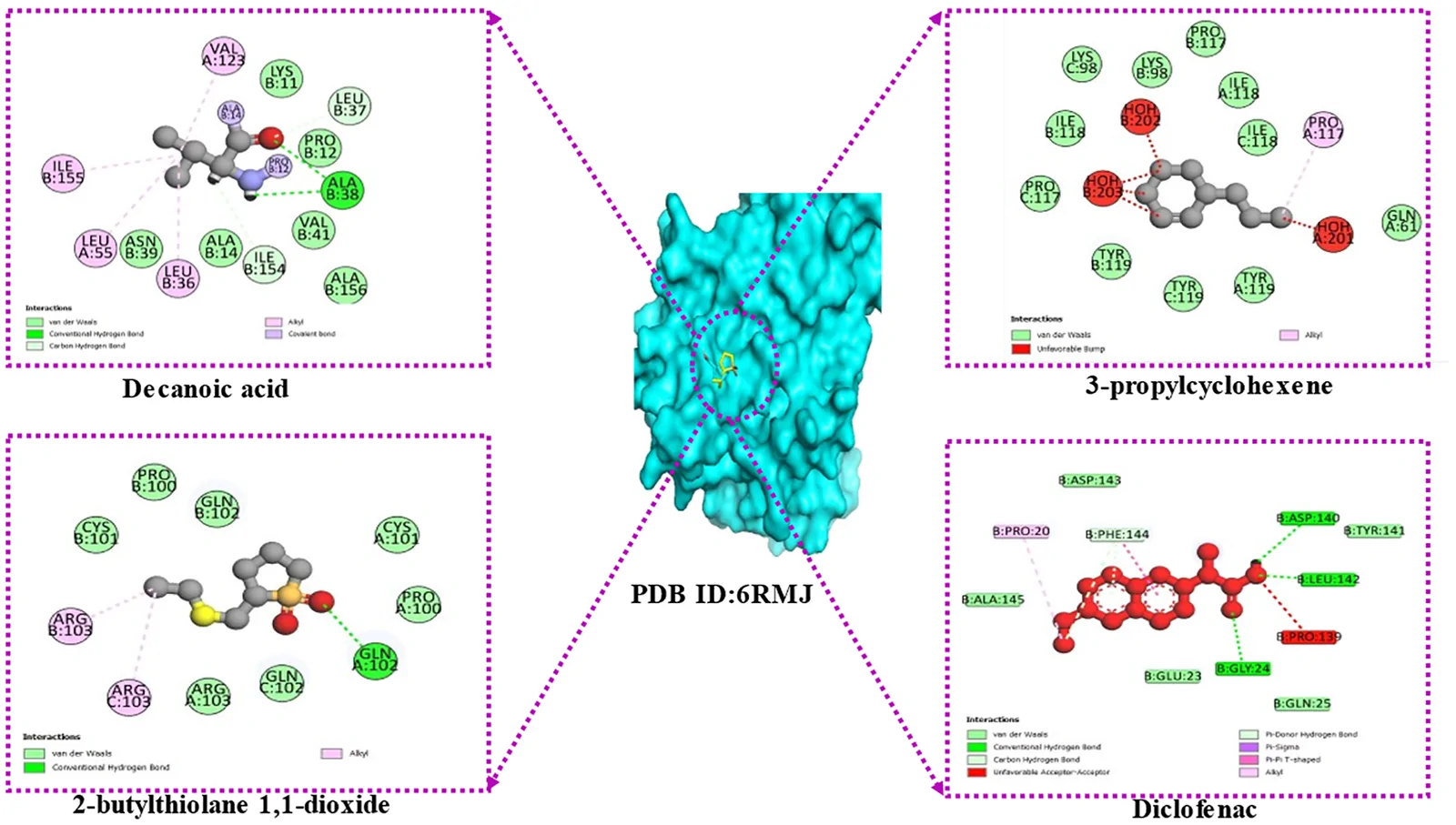
Three-dimensional docking pose of the top-ranked compounds within the active site of the anti-arthritic target protein 6RMJ, illustrating key ligand–protein interactions.

### Molecular docking analysis for analgesic activity.

Molecular docking was used to evaluate the potential analgesic profile of the compound with target protein 5IKR-the results are given below in Table 11. Thus the binding affinity calculated for 3,11-dimethylspiro[5.5]undecane towards this active binding pocket is – 8.7 kcal/mol, which implies it’s quite good as a drug ligand. For comparison purposes the standard analgesic drug Diclofenac displayed a docking score of– 8.3 kcal/mol. Research is ongoing, but it would appear likely that 3,11-dimethylspiro[5.5]undecane fits snugly into the catalytic domain of this target protein channel so as to stabilize ligands result. A detailed list of molecule amino acid residues that influence binding to protein are given in Table 12. Moreover, Fig 16 provides the three dimensional binding pose for ligand in the active binding pocket of protein 5IKR. It shows where the compound sits not only spatially but also: clue about how close it is to various amino acids and what those residues are on which it acts or interacts sufficiently effectively. If we consider the docking results as a whole, 3,11-dimethylspiro[5.5]undecane was found to bind with slightly higher affinity than the reference drug diclofenac. This suggests that computational and pharmacological investigations into novel analgesics might be worthwhile taking as one of their priorities in laboratory research.

**Fig 16:**
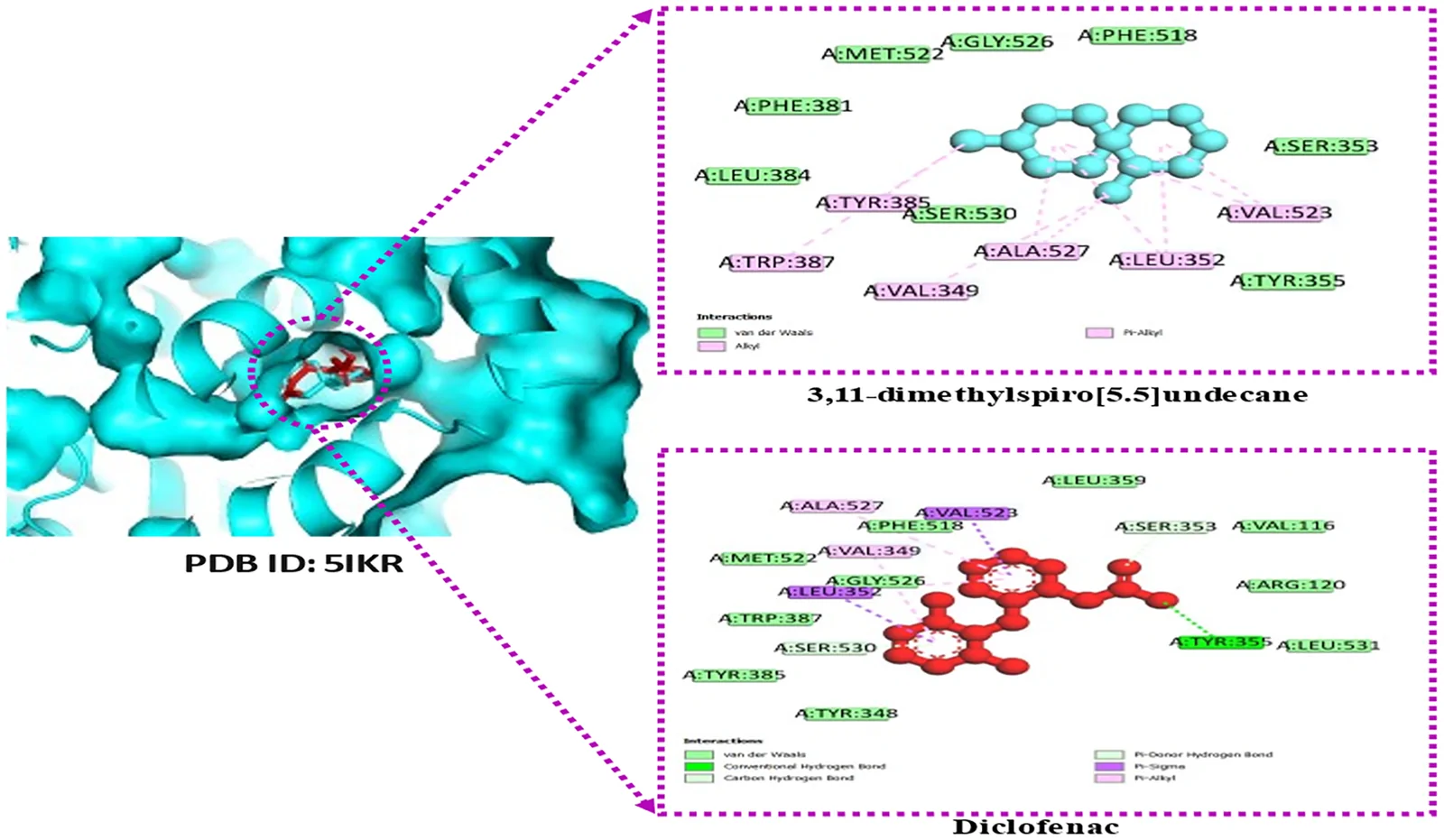
Three-dimensional docking pose of the ligand within the active binding site of the analgesic target protein 5IKR, illustrating key ligand–protein interactions.

### Molecular docking analysis for cytotoxic activity

The cytotoxic potential of the selected phytochemicals was evaluated through molecular docking analysis. The docking scores of the selected compounds against the cytotoxic target protein are summarized in Table 11. Among the ligands examined, 2,3,4-trimethylpentanoic acid has the best docking score of − 8.5 kcal/mol, suggesting that it binds to the active pocket with a favorable interaction. With tetrazolo [1,5 – b] pyridazine we also find a marked binding potential of − 8.3 kcal/mol, which means despite differences in numbers and arrangements it still has comparable or possibly even higher relative proportions for certain reactions than some lower homologues such as amide complexes using these same ligand fragments In addition, methyl 10,13-dimethyltetradecanoate has a somewhat lower but still significant docking score of − 8.0 kcal/mol. These docking scores suggest that the examined compounds are capable of forming stable interactions with the catalytic region of the 1NME protein. Details of the amino acid residues included in compound protein interactions are given in Table 12. Such interactions include hydrogen bonds, hydrophobic contacts and Van der Waals forces, all of which contribute to the stabilization of ligand – protein complexes. The three-dimensional binding configurations of ligands docked into the active site of the protein are shown in Fig 17. Overall, the docking analysis indicates that 2,3,4-trimethylpentanoic acid and tetrazolo [1,5 – b] pyridazine have promising binding affinities towards this cytotoxic protein, suggesting their potential as candidates for further computational and experimental evaluation in cytotoxic drug discovery.

**Fig 17:**
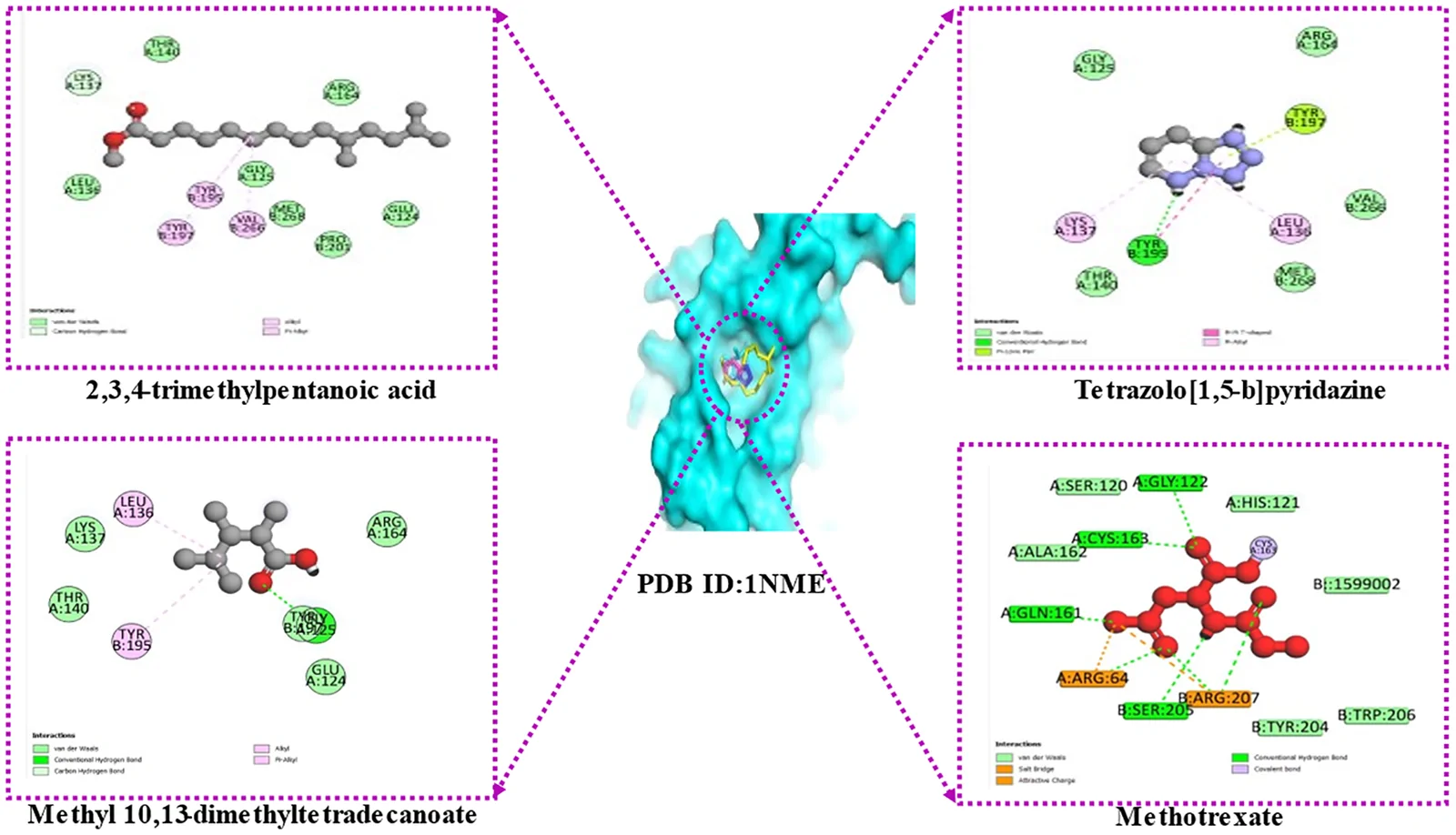
Three-dimensional docking pose of the investigated ligands within the active site of the cytotoxic target protein 1NME, illustrating key ligand–protein interactions.

### Molecular docking analysis for anthelmintic activity

The anthelmintic potential of the selected compound was evaluated using molecular docking analysis against the target protein 1SA0. The docking scores of the test ligand and the reference drug are summarized in Table 11.Among the compounds analyzed, 5-Nitro-2-aminobenzophenone thiosemicarbazone showed the highest binding affinity (−10.6 kcal/mol), indicating a strong interaction within the active site of the protein. In comparison, the standard anthelmintic drug Albendazole exhibited a slightly lower docking score (−9.8 kcal/mol).This higher binding affinity suggests that 5-Nitro-2-aminobenzophenone thiosemicarbazone may interact more effectively with the catalytic region of the target protein, potentially forming a more stable ligand–protein complex. The specific amino acid residues involved in these interactions are detailed in Table 12.Additionally, the three-dimensional binding pose of the ligand within the active site of the 1SA0 protein (Fig 18) provides further insight into its spatial orientation and interactions with surrounding residues.Overall, these findings indicate that 5-Nitro-2-aminobenzophenone thiosemicarbazone demonstrates stronger binding than Albendazole, highlighting its potential as a promising candidate for further computational and experimental studies aimed at developing new anthelmintic agents.

**Fig 18:**
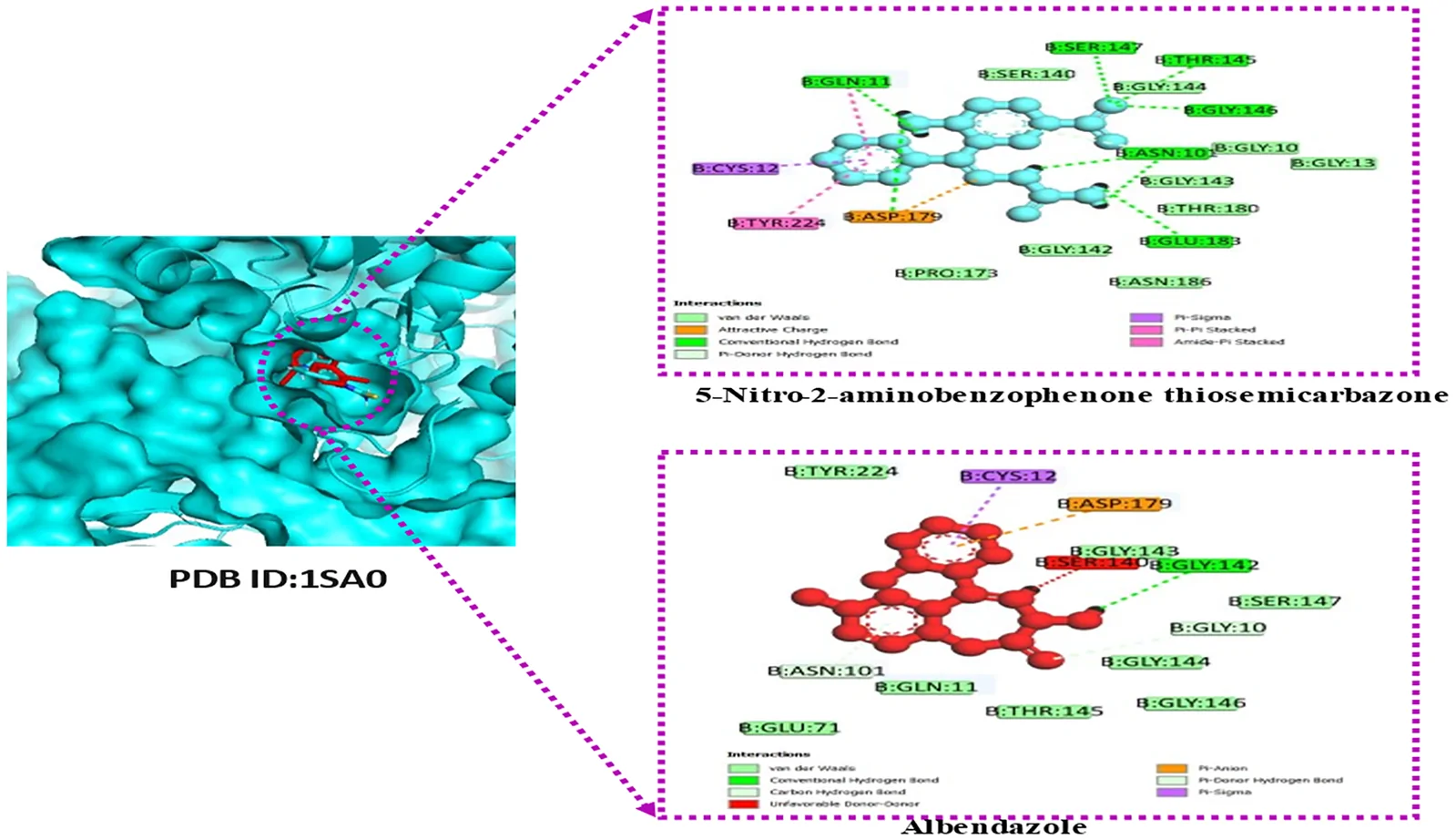
Three-dimensional docking pose of the ligand within the active site of the anthelmintic target protein 1SA0, illustrating key ligand–protein interactions.

### ADMET and drug likeness properties analysis

Selected compounds were subjected to ADMET in silico analysis to determine their favorable pharmacokinetic and toxicological properties. The results have shown that this makes them potential therapeutic agents. The HIA varied from 0.025 to 10.78. The compounds with highest HIA were 12-bromododecanoic acid (4.52) and 2-butylthiolane 1,1-dioxide (10.78). All compounds had low BBB permeability. This suggested a limited brain penetration. Only 2-butylthiolane 1,1-dioxide showed the highest P-gp substrate probability of around 1.56 while other compounds have shown lower P-gp substrate probabilities.

Interactions via cytochrome P450 (CYP) were limited, although tetrazolo[1,5-b]pyridazine and 3-propylcyclohexene exhibit marginally higher IC50 values for CYP2C9 or CYP2C19. All compounds satisfied Lipinski’s rule of five (0.55 < bioavailability score < 0.80) with 3,11-dimethylspiro[5.5]undecane having the highest predicted oral bioavailability (0.80). Low predicted hepatotoxicity (0.147 to 0.607) and high LD50 values (1,800–5,000 mg/kg) indicate an ample safety margin. Compounds like 3,11-dimethylspiro[5.5]undecane and 2-butylthiolane 1,1-dioxide seem promising with good absorption rate and bioavailability coupled with the absence of substantial CYP and hepatotoxicity associated risk factors to promote further investigation as multi-target therapeutic agents data shown in Table 13.

**Table 13:** Predicted ADMET properties of selected compounds, including human intestinal absorption (HIA), blood–brain barrier (BBB) permeability, P-glycoprotein (P-gp) substrate probability, cytochrome P450 (CYP) interactions, Lipinski’s rule compliance, bioavailability score, hepatotoxicity, and median lethal dose (LD50, mg/kg).

Compound name	HIA	BBB	P-Gp Substrate	CYP2C9	CYP2C19	CYP2A4	Lipinski	Bioavailability score	Hepatotoxicity	LD50(mg/kg)
Methyl tetradeca-12,13-dienoate	6.40293	−5.0371	0.294759	0.04903	0.824031	6.691573	0	0.55	0.341389	5000
Tetrazolo[1,5-b]pyridazine	0.025286	−4.66681	−0.14168	0.691518	1.66E-05	0.045286	0	0.65	0.49345	4000
Dibutan-2-yl sulfite	1.645969	−5.09001	0.247082	0.024189		7.990444	0	0.60	0.316156	4250
5-Nitro-2-aminobenzophenone thiosemicarbazone	2.305923	−5.56709	−0.11907	0.995693	0.765252	0.35923	0	0.55	0.206409	3000
3-propylcyclohexene	3.609813	−4.78003	0.139155	0.881796	0.469103	9.21661	0	0.60	0.147418	2500
12-bromododecanoic acid	4.516366	−5.07286	0.17615	0.092538	1.13E-05	3.403239	0	0.55	0.157079	1800
Decanoic acid	2.166186	−4.81923	0.365225	0.910646		8.912218	0	0.70	0.492864	2000
2-butylthiolane 1,1-dioxide	10.77704	−5.01887	1.560685	0.002249		4.718295	0	0.60	0.209717	2500
3,11-dimethylspiro[5.5]undecane	2.705926	−5.01223	0.507706	0.952735		9.055199	0	0.80	0.607333	2000
2,3,4-trimethylpentanoic acid	2.162859	−4.87694	−0.37933	0.178297	1.26E-06	2.513777	0	0.65	0.194416	5000
Methyl 10,13-dimethyltetradecanoate	6.40293	−5.0371	0.294759	0.04903	0.824031	6.691573	0	0.70	0.316156	5000

### Implications of quantum chemical descriptors as drug discovery tools

To further investigate the chemical reactivity and possible biological interactions of the tested phytoconstituents, their frontier molecular orbital (FMO) properties were examined. Table 14 shows the values calculated for EHOMO, ELUMO, energy gap (ΔE), chemical hardness (η) and electronegativity (χ), and Fig 19 shows their electronic distribution of the studied compounds, 2-butylthiolane 1,1-dioxide had the lowest ΔE (ΔE = 0.24909 eV), which suggests that this compound is a more chemically active and more powerful electron donor. In fact, decanoic acid displayed the highest ΔE (0.28050eV) indicating that its molecular structure was the most stable but also least reactive. The moderate energy gaps seen for 3-propylcyclohexene and 2-chloro-1-ethoxy-1-methoxyethane are representative of a good compromise between stability and reactivity, which is ideal for drug-like properties.These were further supported by the chemical hardness values, where decreased hardness relates to increased molecular softness and thus further improved ability to interact with biological targets. In particular, 2-butylthiolane 1,1-dioxide had the lowest η (0.12455 eV), corroborating its highest reactivity and possible binding flexibility. On the other hand, decanoic acid proved to have the most significant hardness (0.14025 eV)due to its stability.Comprehensive analysis of 2-chloro-1-ethoxy-1-methoxyethane (χ = 0.12353 eV) and decanoic acid (χ = 0.12116 eV) showed an improved propensity to act as electron-accepting weble molecules, which likely enhances their interaction with the electron-rich sites of protein targets. These properties are especially important for stabilizing ligand–protein complexes by electrostatic interactions.As a side note, these distribution patterns of the FMO illustrated in Fig 19 reveal that the regions of HOMO are localized above the sites where functional groups are electron-rich, while LUMOs are distributed over electron-deficient centers. The space, thus, provides an environment for ligand binding that assists charge transfer interactions and concomitant molecular recognition and binding affinity strengths.These electronic characteristics highly correlate with the previously achieved in silico molecular docking results and therefore together assist the overall drug discovery process, particularly where compounds in the order of lower ΔE and η were found to bind more strongly with the target protein. Finally, the identified reactivity trends are consistent with the in vitro anti-oxidative and anti-inflammatory activities, implying that electron-donating capacity could be involved in the free radical scavenging and structural maintenance of proteins mechanisms.Moreover, the advantageous tension between reactivity and stability of these compounds supports their possible functionality in vivo, where metabolic stability and target interaction are both necessary. The quantum chemical descriptors correlated collectively reveal that the quantum chemical descriptors are in accordance to general perception regarding the electronic nature of selected phytochemicals being optimal to show biological activity making them promising multi-target therapeutic agents.

**Table 14:** Molecular orbital, chemical hardness, and reactivity descriptor analysis.

Properties	2-butylthiolane 1,1-dioxide	3-propylcyclohexene	Decanoic acid	2-chloro-1-ethoxy-1-methoxyethane
E_HOMO_(eV)	−0.24248	−0.23578	−0.26141	−0.25720
E_LUMO_(eV)	0.00661	0.03503	0.01909	0.01014
∆ E	0.24909	0.27081	0.28050	0.26734
η(Chemical Haedness)	0.12455	0.13541	0.14025	0.13367
χ(Electronegativity))	0.11794	0.10038	0.12116	0.12353

**Fig 19:**
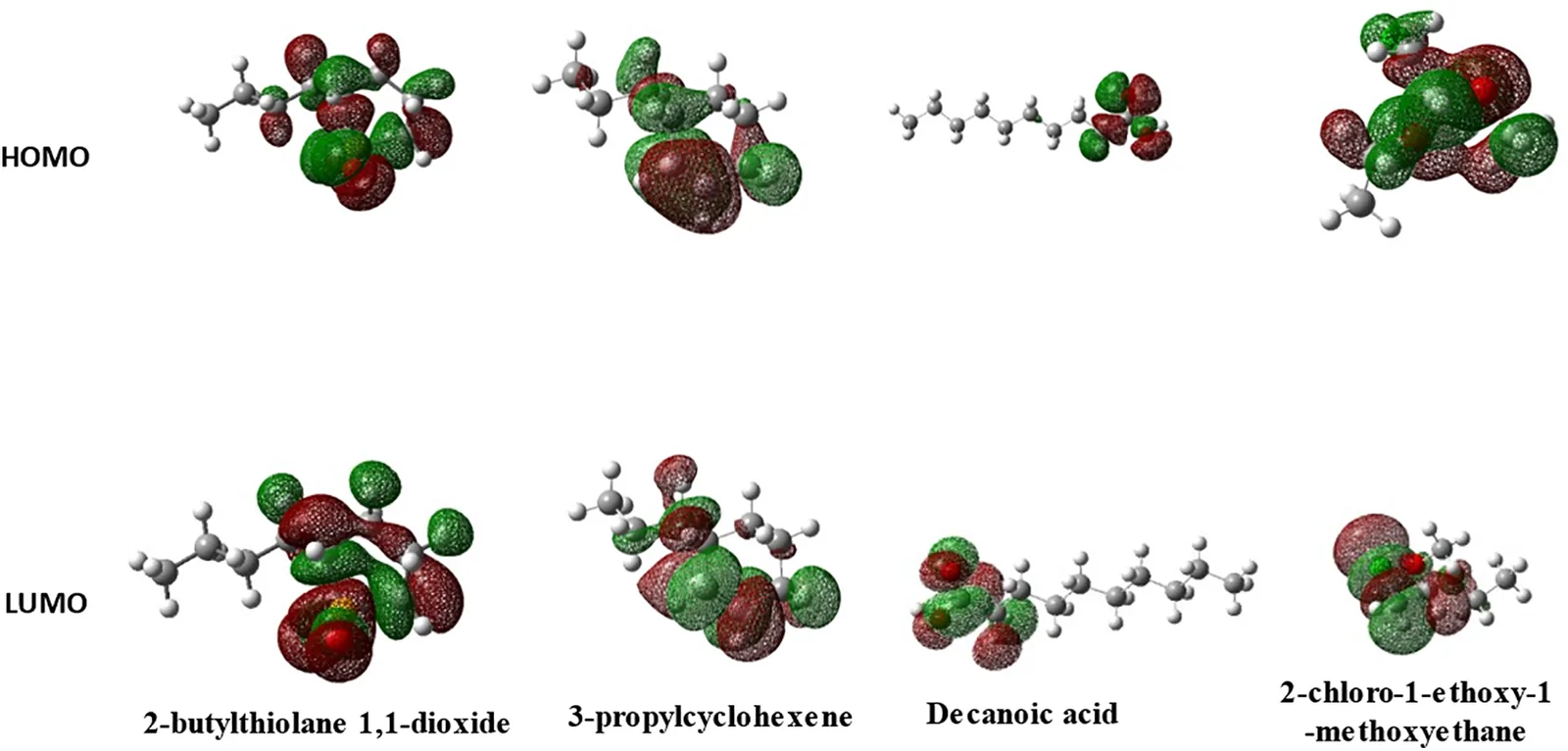
DFT-based molecular orbital and reactivity descriptor profiling of top-ranked compounds [3,11,14,39,64,65].

## Conclusion

The methanolic extract of *Combretum indicum* showed good multidimensional dosing pharmacological potential as evidenced by experimental and computational analysis and it is presented in this study. Mineral profiling demonstrated presence of required elements and absence of unwanted toxic heavy metals reporting its relative safety for therapeutic use. The extract showed significant antioxidant activity, excellent thrombolytic activity higher than standard drug and a strong anti-inflammatory and anti-arthritic activities both in vitro and in vivo. Its wide spectrum bioactivity is also reflected in the presence of significant analgesic, cytotoxic, and anthelmintic activities.

The in silico studies conducted subsequently confirmed these results, indicating strong binding affinities of the candidates against several biological targets as well as ADMET-oriented disposition features and drug-likeness compliance. This was further supported by Density Functional Theory calculation analysis of identified compounds, which revealed the corresponding chemical stability and reactivity, thus indicating a degree of potential as drug candidates.

Collectively, this integrative approach supports the traditional use of *C. indicum* in medicine and highlights its potential as a valuable source of bioactive compounds with multi-target therapeutic promise. The grid box parameters used for molecular docking with AutoDock Vina are provided in S1 Table. Further research involving the isolation, identification, characterization, and clinical validation of its pharmacological activities will be necessary to advance these findings toward new drug development.

## Supporting information

S1 Table The grid box parameters used for molecular docking with AutoDock Vina. (DOCX)
