# Supplementary material for: Integrative in vitro, in vivo, and in silico investigation with mineral profiling of Combretum indicum: A multi-target therapeutic candidate exhibiting antioxidant, thrombolytic, anti-inflammatory, anti-arthritic, analgesic, cytotoxic, and anthelmintic activities
Source: PLoS One. 2026 Oct 26;21(10):e0360173. doi: 10.1371/journal.pone.0360173 (PMC13705604; doi:10.1371/journal.pone.0360173)
Supplement: Supplementary file 1 [file pone.0360173.s001.docx]

### ****Table 1. Grid box parameters used for molecular docking with AutoDock Vina****

| Target Activity | PDB ID | Receptor File | Center X | Center Y | Center Z | Size X (Å) | Size Y (Å) | Size Z (Å) | Exhaustiveness |
| --- | --- | --- | --- | --- | --- | --- | --- | --- | --- |
| Antioxidant | 1DNU | 1DNU.MINIMIZED.pdbqt | 25.7647 | −0.4326 | 13.5217 | 25.0 | 25.0 | 25.0 | 8 |
| Thrombolytic | 2HLO | 2HLO.MINIMIZED.pdbqt | 52.2972 | 92.9279 | 124.6712 | 25.0 | 25.0 | 25.0 | 8 |
| Anti-inflammatory | 1CX2 | 1CX2.fresh_.pdbqt | 42.3318 | 33.5900 | 36.0083 | 25.0 | 25.0 | 25.0 | 8 |
| Anti-arthritic | 6RMJ | 6RMJ-TNF.fresh.pdbqt | −1.8205 | 68.6839 | 127.4897 | 63.9752 | 62.6100 | 25.0 | 8 |
| Analgesic | 5IKR | 5KIR.MINIMIZED.pdbqt | 31.3620 | 8.0353 | 35.3887 | 25.0 | 25.0 | 25.0 | 8 |
| Cytotoxic | 1NME | 1NME.fresh_.pdbqt | 27.9752 | 102.7189 | 11.1137 | 25.0 | 25.0 | 25.0 | 8 |
| Anthelmintic | 1SA0 | 1SA0.FRESH.pdbqt | 82.4102 | 74.5641 | 4.0162 | 25.0 | 25.0 | 25.0 | 8 |
